# Engineering strategies to safely drive CAR T-cells into the future

**DOI:** 10.3389/fimmu.2024.1411393

**Published:** 2024-06-19

**Authors:** Matteo Rossi, Eytan Breman

**Affiliations:** Celyad Oncology SA, Mont-Saint-Guibert, Belgium

**Keywords:** CAR T-cells, cell engineering, gene editing, gene modification, transgene delivery

## Abstract

Chimeric antigen receptor (CAR) T-cell therapy has proven a breakthrough in cancer treatment in the last decade, giving unprecedented results against hematological malignancies. All approved CAR T-cell products, as well as many being assessed in clinical trials, are generated using viral vectors to deploy the exogenous genetic material into T-cells. Viral vectors have a long-standing clinical history in gene delivery, and thus underwent iterations of optimization to improve their efficiency and safety. Nonetheless, their capacity to integrate semi-randomly into the host genome makes them potentially oncogenic via insertional mutagenesis and dysregulation of key cellular genes. Secondary cancers following CAR T-cell administration appear to be a rare adverse event. However several cases documented in the last few years put the spotlight on this issue, which might have been underestimated so far, given the relatively recent deployment of CAR T-cell therapies. Furthermore, the initial successes obtained in hematological malignancies have not yet been replicated in solid tumors. It is now clear that further enhancements are needed to allow CAR T-cells to increase long-term persistence, overcome exhaustion and cope with the immunosuppressive tumor microenvironment. To this aim, a variety of genomic engineering strategies are under evaluation, most relying on CRISPR/Cas9 or other gene editing technologies. These approaches are liable to introduce unintended, irreversible genomic alterations in the product cells. In the first part of this review, we will discuss the viral and non-viral approaches used for the generation of CAR T-cells, whereas in the second part we will focus on gene editing and non-gene editing T-cell engineering, with particular regard to advantages, limitations, and safety. Finally, we will critically analyze the different gene deployment and genomic engineering combinations, delineating strategies with a superior safety profile for the production of next-generation CAR T-cell.

## 1 Introduction

In the last decade, immune cell therapy, and in particular the introduction of chimeric antigen receptor (CAR) T-cells, reprogrammed immune cells expressing a CAR to specifically target tumor antigens, has left an outstanding mark in oncological research and clinical practice, revolutionizing the way we conceive cancer therapy. Unprecedented results, with complete responses as high as >90%, were achieved in several hematological malignancies such as advanced or resistant large B-cell lymphoma, acute lymphoblastic leukemia, and multiple myeloma (1, 2).

Despite the initial successes scored by immune cell therapy, the limitations of the approach are steadily becoming clearer. The autologous T-cell collection from patients in sufficient number and quality for manufacturing purposes can be difficult, due to the underlying disease and to prior therapies, and the current vein-to-vein time for CAR T-cell products can be incompatible with the status of patients with aggressive and fast-progressing disease. Moreover, further challenges need to be overcome in solid tumor indications. Homogeneously expressed tumor antigens that are not shared by critical healthy tissues are difficult to find, and the harsh conditions of the tumor microenvironment (TME), including chronic antigen stimulation, insufficient co-stimulation, low pH, limited oxygen and nutrients, toxic metabolites, and immunosuppressive factors, limit CAR T-cell homing and migration and induce exhaustion (3). It is therefore clear that further improvements are needed to expand the range of applicability of CAR T-cells and to achieve satisfactory results in other indications.

Many of the aforementioned limitations could be tackled by acting upon two key parameters: the delivery methodology of the transgene and the engineering strategy used for the improvement of the CAR T-cell therapy.

**Figure 1:**
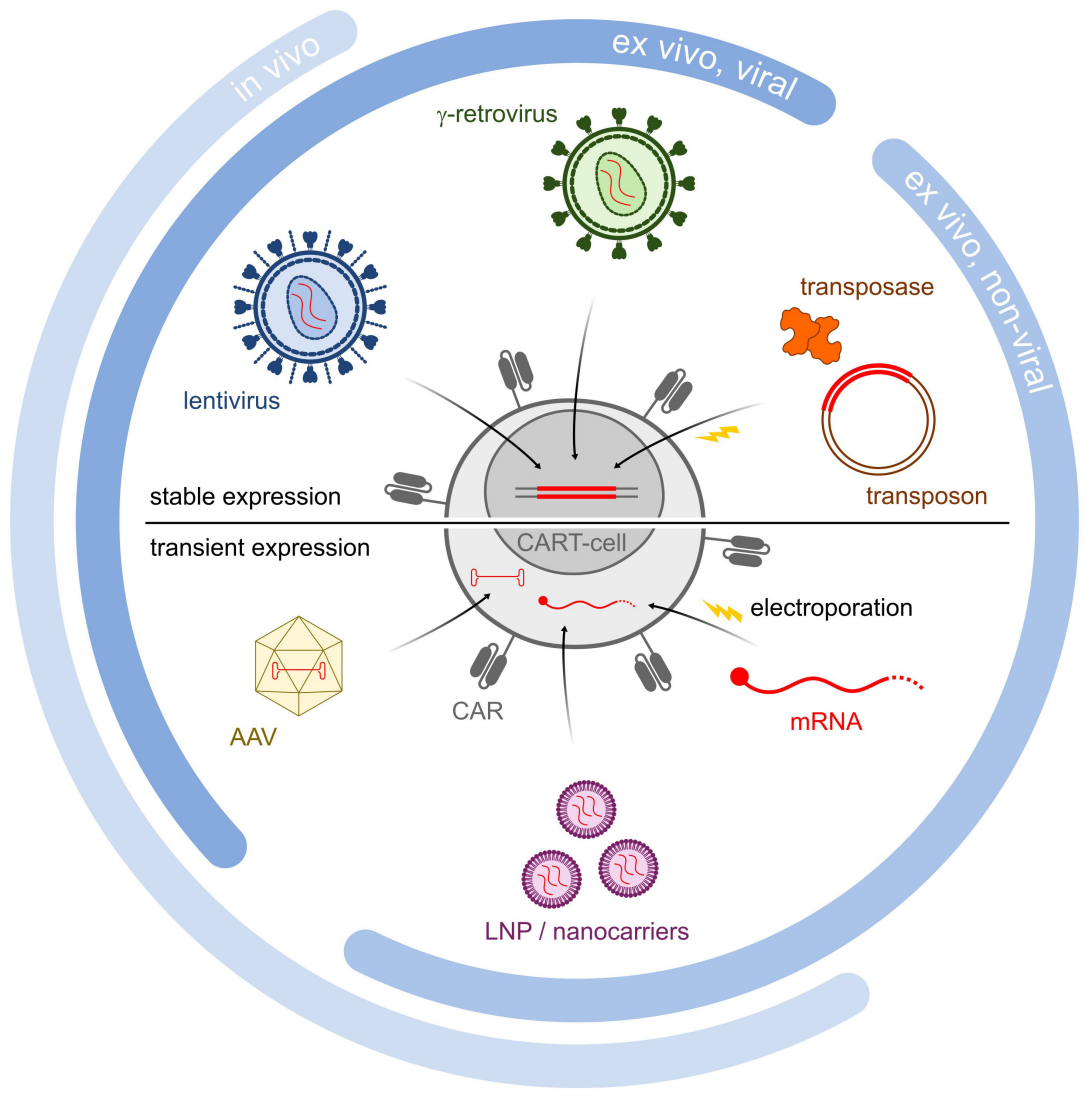
Schematic illustration of different gene delivery methods. Methodologies are divided into *in vivo*, *ex vivo* viral and *ex vivo* non-viral methodologies. Lentiviruses, retroviruses and transposons all are incorporated into the genome, and lead to stable CAR expression (*upper half*). In contrast, AAV LNP/Nanocarriers and mRNA all lead to transient CAR expression (*lower half*).

**Figure 2:**
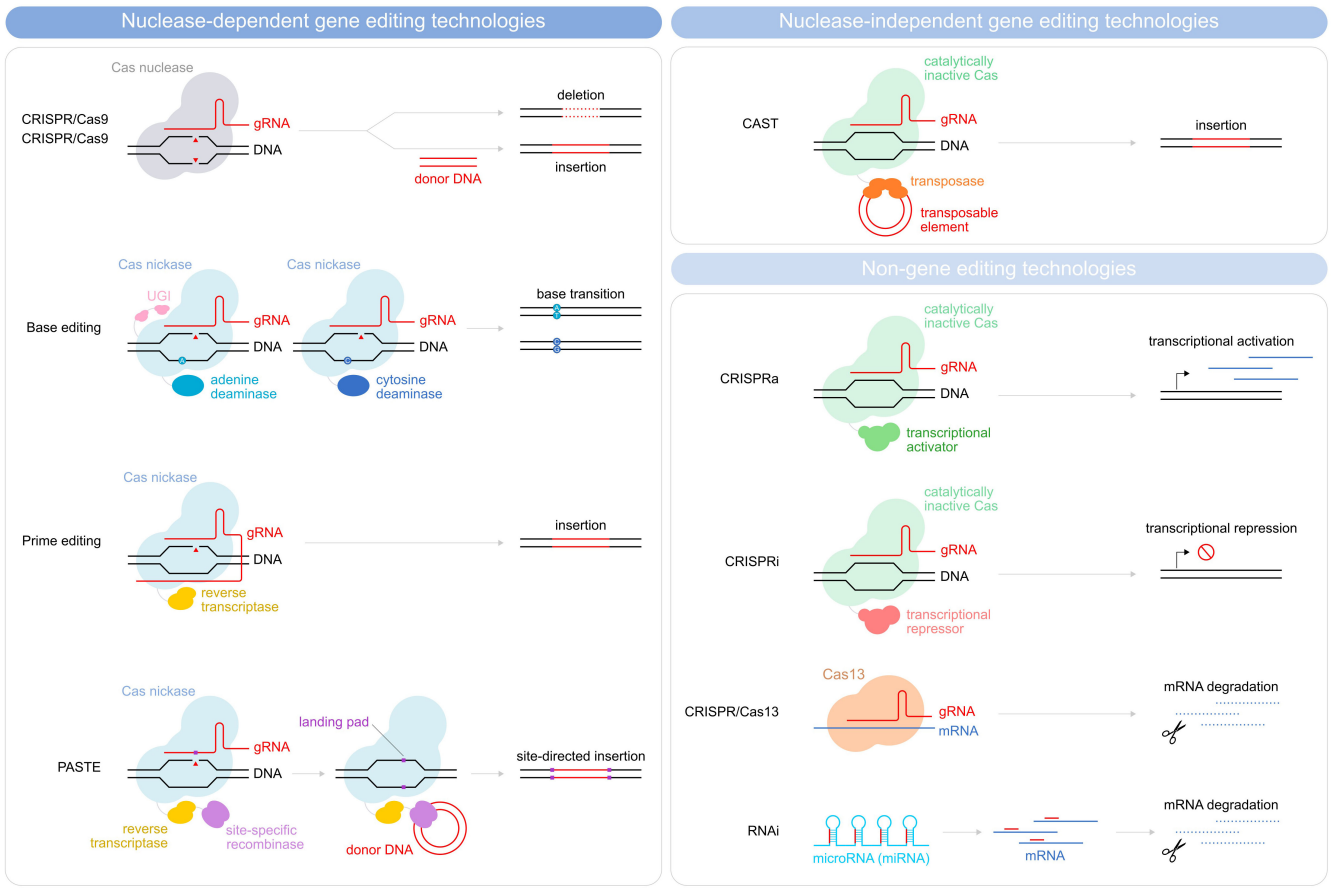
Schematic illustration depicting methods for gene editing and non-gene editing technologies. Gene editing strategies used in CAR T-cells depicted are divided into nuclease-dependent gene-editing technologies (CRISPR/Cas9, Base editing, prime editing, and PASTE) and nuclease-independent gene editing technologies (CAST). Non-gene editing technologies (CRISPRa, CRISPRi, CRISPR/Cas13, and RNAi) are also shown.

Indeed, optimizing criteria such as the efficiency and stability of transgene expression, the genetic cargo capacity, the scalability, and the production costs would improve the manufacturability of the CAR T-cells and allow for more extensive manipulation. For the purpose, a variety of approaches are currently being evaluated for transgene delivery (**Figure 1**, **Table 1**), both vector-based (γ-retroviruses, lentiviruses) and non-vector-based (transposons, nanovectors, mRNA). Likewise, engineering the CAR T-cells beyond the mere CAR introduction may help improve both their manufacturability and functionality. A typical example is the ablation of key T-cell surface markers, such as the T-cell receptor and the Human leukocyte antigens (HLAs) to generate allogeneic, off-the-shelf CAR T-cells, and thus make the therapy more easily and broadly available. Another sought-after engineering goal, aimed at providing better resistance and performance of the CAR T-cells in the TME, is the elimination of negative regulators of the T-cell function (e.g. receptors for immune checkpoint or for inhibitory cytokines). Many different engineering strategies are being developed to improve these as well as other CAR T-cell characteristics (**Figure 2**, **Table 2**). In particular, gene editing, and especially clustered regulatory interspaced short palindromic repeat/associated nuclease protein 9 (CRISPR/Cas9)-based approaches, made genetic engineering faster, easier, and more versatile than ever (4).

**Table 1:** Advantages, limitations, and safety risks associated with the different transgene delivery systems.

	Viral delivery systems		Non-viral delivery systems			*In vivo* delivery systems		
	γ-retroviruses	Lentiviruses	Transposons	Nanovectors	mRNA	Lentiviruses	AAVs	LNPs, NCs
Transfer method to the target cells	Transduction (*ex vivo*)	Transduction (*ex vivo*)	Electroporation	Electroporation	Electroporation, cationic lipids or polymers	Transduction (*in vivo*)	Transduction (*in vivo*)	Endocytosis
Efficiency	High	High	Moderate to low	Moderate	High	High	High	High
Cargo size	Limited (<10 kb)	Limited (<10 kb)	Large (~14 kb, >100 kb with BACs)	N/A	N/A	Limited (<10 kb)	Limited (<4 kb)	N/A
Integration	Semi-random	Semi-random	Random (SB), semi-random (PB)	No	No	Semi-random	No	No
Stability of gene expression	High	High	High	Transient	Transient	High	Transient	Transient
Immunogenicity	N/A	N/A	N/A	N/A	N/A	Low	High	Very low
Manufacturing complexity	High	High	Moderate	Low	Low	High	High	Low
Manufacturing costs	High	High	Moderate	Low	Low	High	High	Low
Clinically evaluated for CAR T-cell generation	Yes	Yes	Yes	No	Yes	No^1^	No^1^	No
Theoretical risk of genotoxic effects	Yes	Yes	Yes	Extremely low	No	Yes	Extremely low	Extremely low
Reported genotoxic effects in the clinics	Yes	Yes	Yes	N/A	No	Yes	No	N/A

^1^ Clinically evaluated for gene therapy applications.

AAVs, adeno-associated viruses.

LNPs, lipid nanoparticles.

NCs, nanocarriers.

BACs, bacterial artificial chromosomes.

SB, Sleeping Beauty.

PB, Piggy Bac.

**Table 2:** Advantages, limitations, and safety risks associated with the different CAR T-cell engineering technologies.

	Gene editing technologies					Non-gene editing technologies		
	ZFN, TALEN	CRISPR/Cas9	Base editing	Prime editing	PASTE	CRISPRa, CRISPRi	CRISPR/Cas13	RNAi
Applicability to CAR T-cell engineering	Complex	Complex	Complex	Complex	Extremely complex	Moderate	Moderate	Easy
Clinically validated	No	Yes	No	No	No	No	No	Yes
Clinically evaluated for CAR T-cell generation	Yes	Yes	Yes	No	No	No	No	Yes
Multiplexing	Yes, difficult	Yes, difficult	Yes, difficult	Yes, difficult	N/A	Yes	Yes	Yes
Efficiency in CAR T-cells	Low	Good, but decreases with the number of targets	Low	Low	Low	High	High	High
Risk of genotoxic effects	High, increases with the number of targets	High, increases with the number of targets	Moderate	Moderate	Moderate	Extremely low	No	No
Risk of off-target effects	Moderate	Moderate	High	High	Low	Moderate	High	Low

Novel CAR T-cell products are therefore emerging that rely on different combinations of transgene delivery systems and engineering approaches. This may lead the immune cell therapy field to advance toward new therapeutic successes, but at the same time it opens new outstanding questions about the clinical scalability, efficacy, and safety of these products. Safety in particular warrants special attention, as genotoxicity may occur, and has already been reported in some instances, both due to the delivery system and the engineering technology. Here, we will review the vectors and engineering tools used for CAR T-cell production at the clinical and preclinical level, as well as the up-and-coming approaches that are being explored for next-generation products, outline the advantages and limitations each of them has, and discuss which combinations may be exploited to design safer and more effective immune cell therapies.

## 2 Transgene delivery systems

### 2.1 Retroviral-based vectors

Retroviral-based vectors are the preferred gene delivery system for the generation of CAR T-cells (5). Indeed, all U.S. Food and Drug Administration (FDA)-approved CAR T-cell therapies are engineered using vectors derived either from γ-retroviruses (Yescarta, Tecartus) or lentiviruses (Kymriah, Breyanzi, Abecma, Carvykti), two of the seven members of the Retroviridae family (6, 7).

Retroviruses are lipid-enveloped viruses with a single-stranded diploid RNA genome. Upon infection, the viral genomic RNA is retrotranscribed by an RNA-dependent DNA polymerase (reverse transcriptase) and integrates into the host genome thanks to specific sequences in the long terminal repeats (LTR) flanking the viral genome. The U3 region of the LTR also acts as a promoter/enhancer to drive the transcription of the viral genes. All retroviruses require the same basic elements to assemble the viral particle, although slightly different isoforms are used by γ-retroviruses and lentiviruses: *gag*, encoding for the capsid proteins, *pol*, for the reverse transcriptase and other factors necessary for integration, and *env*, for the envelop glycoprotein, which determines the virus tropism. In the case of lentiviruses, the *rev* gene is also required to enhance the nuclear export and expression of the other transcripts (8).

The ability to efficiently deliver a cargo of up to 8–10 kb (6, 8), the weak immunogenicity (9, 10), and the capacity to integrate their genetic information into the DNA of the host cells (6, 8) make retroviral-based vectors especially suitable for gene delivery, ensuring a relatively high integration efficiency and long-term stable transgene expression.

#### 2.1.1 Safety considerations on retroviral-based vectors

The overall safety and efficacy of retroviral vectors for clinical applications has been proven in many clinical trials (reviewed in (11, 12)), first in the gene therapy space and more recently in immune cell therapy. Still, several potential risks remain associated with the use of retroviral vectors in the clinics.

One obvious safety concern inherently linked to the use of viral vectors is that recombination of the vector with wild-type viruses may lead to the unintended generation of replication-competent viral particles, during vector or CAR T-cell manufacturing or in the recipient patients. Thanks to the long-standing preclinical and clinical experience with viral vector delivery in gene therapy, this risk has been greatly mitigated by optimizing the packaging system design. Indeed, in current third-generation retroviral packaging systems, the genes required for vector production are split across three different plasmids: one plasmid carries the engineered viral genome, containing the transgene(s) and only the viral sequences necessary for packaging and integration, whereas all the other components are supplied *in trans* by a second plasmid providing the structural *gag* gene and the regulatory proteins, and by a third plasmid providing the *env* protein. Such design ensures that, even in case of a recombination event between the engineered vectors and the genome of wild-type viruses, the resulting genetic material will never carry all the essential viral genes and therefore that no replication-competent viral particles can emerge. Safety can be further increased by introducing deletions in the U3 region of the LTR, which functions as promoter/enhancer for the viral genome, thus making the viral vector self-inactivating (SIN) upon integration (13). Most of the lentiviral vectors used in clinical applications are third-generation SIN vectors (14), ensuring a higher safety profile, although they pose alternative issues during manufacturing, as the LTR deletions tend to reduce the vector titers (15), and thus require further tweaking of the manufacturing conditions, such as optimization of the packaging cell lines, of the transduction efficiency and of the purification steps. At the regulatory level, the U.S. Food and Drug Administration (FDA) requires replication-competent retroviruses (RCRs) and lentiviruses (RCLs) detection assays for viral vectors and virally transduced cell products (16), and many CAR T-cell clinical trial protocols establish patient testing for preexisting viral infections as a requirement.

A second risk associated with CAR T-cell manufacturing with viral vectors is the unintentional introduction of the transgene in other cell types. Viral vectors are pseudotyped, i.e., the original *env* glycoprotein is replaced to improve the tropism for different primary cell types. Lentiviral vectors, in particular, commonly use the vesicular stomatitis virus glycoprotein (VSV-G), which recognizes the ubiquitously expressed low-density lipoprotein (LDL) receptor (14). Pseudotyping with VSV-G allows the transduction of a wide range of cells, but at the same time increases the chance of off-target transduction. Development of a resistant transgenic leukemia clone has already occurred in a patient treated with the CAR T-cell therapy Kymriah, manufactured with a VSV-G pseudotyped lentiviral vector, upon the accidental transduction of a leukemia B cell during production (17). Although rare, such events highlight the risk of using integrating vectors with broad tropism. As alternatives, the introduction of more stringent purification steps during manufacturing or the use of more specific *env* glycoproteins, such as a measles envelope-based chimeric protein capable of targeting the CD3 receptor only present on T-cells (18), have been proposed.

A third outstanding point is whether CAR integration in the T-cell genome can affect the safety and efficacy of CAR T-cell therapy. Despite the integration of retroviral vectors being not targeted, it is not a random process. Rather, it has been defined as semi-random, as different genomic features are diversely susceptible (19, 20): vector integration preferentially occurs at fragile sites, transcriptionally active regions and those recurrently involved in translocation events (21–26). γ-retroviruses and lentiviruses exhibit a distinct pattern of integration within the host genome (27), which is maintained in the derivative gene delivery systems (28) and has been confirmed in CAR T-cells generated with these technologies (29). γ-retroviral vectors preferential insert near transcription start sites, CpG islands, enhancers, and promoters. Given the promiscuous nature of the γ-retroviral LTR enhancer/promoter, insertion close to regulatory elements can lead to induction of neighboring host genes. If proto-oncogenes are involved, this may result in oncogenic transformation: in two distinct clinical trials for the treatment of inherited immunodeficiencies, patients injected with γ-retrovirus-engineered hematopoietic stem cells developed leukemia as a result of insertional activation of proto-oncogenes *LMO2*, *MDS1*-*EVI1*, *PRDM16* and *SETBP1* (30, 31). Lentiviral vectors, on the other hand, integrate more frequently within transcription units, preferentially in introns of transcriptionally active genes. Such integration pattern, along with the frequent use of eukaryotic promoters in SIN vectors to replace the inactive LTR promoter/enhancer, reduces the risk of insertional oncogenesis for lentiviral vectors (32). Still, the site of CAR integration may affect gene expression at the transcriptional level, by leading to loss-of-function mutations, or at the post-transcriptional level, by impairing alternative splicing (29). These alterations can in turn impact CAR T-cell function and, as a consequence, the therapeutic outcome. In a genome-wide analysis of retroviral vector integration on pre-infusion CAR T-cell products, poor clinical response was associated with more integration events in genes involved in neutrophil activation (29), which may mediate immune suppression activity (33, 34). Likewise, in patients experiencing high-grade cytokine release syndrome (CRS) insertions were most commonly found in pathways involved in acetyltransferase/transmembrane transporter activity (29). In a leukemia patient, complete response was driven by the profound expansion of a single CD19 CAR T-cell clone (35). The unusual clonal expansion was linked to loss of TET2 activity: the patient carried a missense mutation in one allele of the *TET2* locus, and the CAR transgene had integrated into the functional *TET2* allele, thus abolishing TET2 function (35). Similarly, clonal expansion of a CD22 CAR T-cell upon integration of the transgene in the *CBL* locus was observed prior to eradication of residual disease in a patient with B-cell acute lymphoblastic leukemia (36). Although in the last two examples the insertional mutagenesis events resulted in the expansion of therapeutically effective clones, insertion in undesirable loci may stochastically lead to less favorable outcomes. As of December 31, 2023, the FDA has reports 22 cases of secondary T-cell cancers in patients treated with five of the six FDA-approved CAR T-cell products (37). Among the 14 cases for which adequate data were available, half have manifested within 1 year from administration (range: 1 to 19 months), and in three cases the CAR transgene has been detected in the malignant clone, suggesting a direct involvement of the CAR T-cell product in the development of the secondary malignancy. With more than 27,000 doses of administered only in the United States to date, the overall rate of CAR T-cell-related secondary cancers is still quite low. However, the diffusion of CAR T-cell therapies, particularly for indications outside oncology (38–42), calls for particular attention to the matter. Along this line, the FDA has recently issued a draft guidance recommending the long-term monitoring for adverse events, including cancer, of patients receiving CAR T-cell products engineered with integrating vectors (43).

### 2.2 Non-viral gene delivery

Despite their widespread use, the application of viral vectors for CAR T-cell generation is subjected to several limitations. Safety concerns linked to the risk of insertional mutagenicity are emerging and, as a consequence, the regulatory constraints are becoming increasingly complex. Moreover, the payload size, although sufficient for classic CARs, may become limiting for more advanced designs requiring the expression of other elements (e.g., armored CAR T-cells, dual CARs, or tandem CARs) (44). From the manufacturing standpoint, large-scale GMP-grade viral vector production involves intricate production, purification, and quality assessment steps and is costly. Hence alternative, non-viral delivery methods for the generation of CAR T-cells are actively being explored, such as transposons, nanovectors and integration-deficient viral vectors.

#### 2.2.1 Transposon-based delivery systems

Among non-viral gene delivery methods, transposon-based systems are the most advanced and promising in preclinical and clinical settings. Transposons are mobile genetic elements with the ability to reposition themselves within the genome (45, 46). Classically, transposons encode for a transposase gene flanked by inverted terminal repeats (ITRs). The transposase recognizes and binds sequences into the ITRs, catalyzes the excision of the transposon from its original position, and integrates it into another chromosomal locus, without the need for sequence homology. Transposon-based delivery systems have been designed by splitting the transposase function and the ITRs into two components, with the payload lying between the two ITRs in a transposon vector or minicircle, and the transposase supplied in *trans*. The most widely used transposon delivery systems are the Sleeping Beauty (SB), reconstructed from inactive transposon sequences isolated from fish genomes (47) and the first transposon shown capable of efficient transposition in vertebrate cells, and the PiggyBac (PB), originally identified in insect cell lines (48). The two systems have many common characteristics and advantages. They allow permanent genomic insertion of transgene cassettes, leading to sustained and efficient transgene expression. Opposed to retroviral vectors, that undergo a severe loss of vector titer for payloads above ~9 kb, they have less strict constrains on the cargo size: the PB system can accommodate up to ~14 kb, and the SB has been pushed to over 100 kb when in combination with bacterial artificial chromosome (BACs) (49). The transposon elements can be maintained and propagated as plasmid DNA, making them simple and inexpensive to manufacture, with estimated costs 5 to 10 times lower than the viral vector production (50). Moreover, transposon systems efficiently transfect resting and naïve primary T-cells, not requiring T-cell activation as a prerequisite for gene delivery during CAR T-cell manufacturing, and thus potentially leading to products with superior phenotypical characteristics (51). As such, transposon-based gene delivery systems maintain the favorable characteristics of integrating viral vectors (i.e., stable chromosomal integration and long-lasting transgene expression) while bypassing many of their shortcomings (15).

#### 2.2.2 Limitations and advancements of transposon-based delivery systems

Although the greater cargo capacity remains one of the main advantages of transposons over viral delivery systems, an inverse correlation between the size of the payload and the efficiency of the transposition has been observed, and overall transposons display lower transfection efficiencies than viruses (52, 53). This is partially linked to the technology most widely used to deliver the transposon components into the target cells. Indeed, the primary method of non-viral gene delivery is through electroporation, i.e. the application of electrical fields to cells to generate pores in the cell membrane, allowing the entrance of the exogenous material. This technique, however, can cause high stress to the recipient cells, resulting in decreased viability and cell loss (54–56). As one of the main factors determining the magnitude of the damage is the amount of DNA delivered, the electroporation toxicity can be mitigated by reducing the size of the transposon vector, e.g. by using minicircles (57), and by delivering the transposase in other forms than plasmid DNA, e.g. mRNA or protein (58). These alternatives also grant a higher level of safety over the delivery as DNA, due to the transient expression of the enzyme and the impossibility of integration of the transposase-coding sequence into the host genome, thus preventing the repeated and unintentional mobilization of the transposon. In parallel, transposases have also been extensively optimized, increasing their transposition efficiency (59–62), to make CAR T-cell production through this method more scalable (60). In particular, the current benchmark SB transposase, SB100X (59), allows for viral-vector-like efficiency of gene integration, thanks to a 100-fold increase in transposition activity in comparison to the first-generation enzyme. Such improvement comes from the combination of molecular evolution and rational, crystal structure-driven optimization of the DNA binding domain through point mutation (61). Through a similar approach, the SB100X transposase has been further engineered to overcome the stability, solubility, and aggregation issues that limited its use as recombinant protein in CAR T-cell manufacturing (58). The improved biochemical properties of this high solubility SB (hsSB) enable purification of biologically active recombinant protein, electroporation of the protein into human cells, and freeze-thaw cycles without compromising transposase activity. As a result, anti-CD19 CAR T-cells could be efficiently generated via hsSB electroporation and displayed antitumor potency in xenograft mice comparable to approved viral vector-based commercial products (58).

#### 2.2.3 Safety considerations on transposon-based delivery systems

In the last decade, an increasing number of clinical trials has been launched using CAR T-cells generated via transposon-based delivery systems (extensively reviewed in (15, 63, 64)), with efficacy results in line with trials based on retroviral-vector generated CAR T-cells. Unfortunately, however, the same kind of safety issues have also emerged. In the CARTELL trial (ACTRN12617001579381), a phase-I study investigating the efficacy and safety in relapsed and refractory B-cell malignancies of an anti-CD19 CAR T-cell product generated with the PB technology, 2 out of 10 patients developed CAR T-cell-originated lymphoma, resulting in one fatality (65). *Post-hoc* analysis revealed an unusually high vector copy number (VCN) of the transgene (24 in one patient, compared to the FDA-recommended threshold of 5) in the malignant CAR T-cells, associated with significant copy number gains and losses of multiple chromosomes and transcriptional readthrough from the transgene promoter, although the other patient only showed a VCN of 4 (66). Interestingly, intronic insertion into the *BACH2* gene, with consequent downregulation of the gene expression, was observed in both patients. BACH2 is a DNA-binding transcriptional regulator with a putative tumor suppressor role and has already been associated with cutaneous T-cells lymphomas (67, 68). Although no causal correlation could be established between these events and the CAR T-cell-originated malignancies in exam, it is conceivable that a high VCN and the integration into or in the vicinity of proto-oncogenes increase the probability of insertional oncogenesis (64).

Conversely, no adverse events were observed in clinical trials where CAR T-cells were generated via SB technology. The reason may reside in the different integration profiles of the two transposon-derived vectors. Indeed, PB displays a γ-retrovirus-like integration pattern, with a higher frequency of insertion into transcriptional start sites of genes, CpG islands and DNase I hypersensitive sites (69, 70), and it is more prone than SB to associate with oncogenes (71). Conversely, SB integration occurs in a close-to-random manner (69, 72), so that SB has a higher probability to land in safe harbor sites compared to PB and retroviral vectors (69).

Another safety liability of the PB system may be the recent discovery in the human genome of an active DNA transposase with high homology to the PB enzyme, namely piggyBac transposable element derived 5 (PGBD5) (73, 74). PGBD5 catalytic activity has already been mechanistically linked to site-specific DNA rearrangements associated with several childhood solid tumor types (75). PGBD5 can possibly mediate the remobilization of PB transposons in PB-engineered human cells, although some observations suggest that PGBD5 may not be able to efficiently bind, excise or integrate the PB transposon, due to species-restricted recognition of the cognate ITRs (76).

Based on the available information, despite both transposon-based systems overall represent a valid alternative to viral vectors for CAR T-cell manufacturing, SB may have an edge over PB and viral vectors themselves thanks to its more favorable safety profile. Still, it is important to remark that any technology based on the integration of genetic material into the host genome presents an inherent risk of causing adverse events due to gene dysregulation or insertional mutagenesis.

#### 2.2.4 Non-integrating delivery systems

Approaches that do not require the stable integration of the CAR-encoding transgene may overcome the risks associated with integration. Potential alternatives under preclinical investigation are non-viral episomal DNA nanovectors, such as the nano-S/MARt (nS/MARt) (77), and integration-deficient viral vectors (78, 79). Despite proof of concept for the generation of clinical-grade CAR T-cells using these technologies has been obtained, the therapeutic efficacy of such products still awaits clinical validation. Another strategy to eliminate the risk of oncogenic insertion is to transiently express the CAR from an mRNA template (80). The CAR-encoding mRNA, typically delivered into T-cells by electroporation, ensures transgene expression for approximately one week, with expression levels declining over time (80, 81). Such transient expression may also be a valuable means to reduce potential toxicity, particularly when the CAR target is also present in healthy tissues. The available clinical data suggest that mRNA-generated CAR T-cells have a good safety profile and exhibit short-term anti-tumor efficacy (82, 83), although by means of multiple injections, but their ability to achieve durable responses has yet to be proven.

### 2.3 *In vivo* CAR T-cell therapy

The next frontier of immune cell therapy manufacturing may completely eliminate *ex vivo* cell manipulation, and rather aim at the generation of the therapeutic cells directly *in vivo* (84). Indeed, the possibility of selectively delivering the genes of interest – the CAR components in this specific case – to the target cells directly within the patient’s body would overcome in a single leap most manufacturing and logistic hurdles that currently limit the availability and diffusion of immune cell therapies.

In the last decade the toolbox for gene delivery has been further developed to meet the needs of immune cell therapy, with particular regard to the specificity of the vector targeting, in order to minimize toxicity linked to high vector doses and off-target effects. Target specificity was mainly pursued by giving the vectors selectivity for immune cell markers, like CD3, CD4, CD5, or CD8, through the use of high-affinity binders, such as scFvs or designed ankyrin repeat proteins (DARPins) (85). These advancements supported the successful *in vivo* generation of CAR T-cells in preclinical models.

The most widely used *in vivo* delivery systems to date are lentiviral vectors, adenovirus-associated vectors (AAVs), and non-viral vectors such as lipid nanoparticles (LNPs) and nanocarriers (NCs).

#### 2.3.1 Application of lentiviral vectors to *in vivo* CAR T-cell generation

VSV-G pseudotyped lentiviral vectors have a broad tropism, achieving high transduction efficiencies on different human cell types. A major risk for *in vivo* delivery is the unintentional engineering of non-target cells, as no selection and purification process of the recipient cells is possible. Attempts to alter receptor usage of the VSV-G protein have been made, e.g. by fusing to the VSV-G antibody single-chain variable fragments (scFv) specific for surface markers expressed on the target cells.Lentiviral vectors have been successfully targeted towards CD30 and epidermal growth factor receptor (EGFR) (86), but the engineering of VSV-G remains challenging, as it mediates both receptor binding and membrane fusion (87). A more viable strategy relies on the substitution of VSV-G with glycoproteins from alpha- and paramyxoviruses, which have separate envelope proteins for binding and fusion and thus allow for alterations of the tropism without interfering with the fusion process (88, 89). Such lentiviral vectors have been targeted against T-cell markers (CD3, CD4, CD8) using both scFvs and DARPins (reviewed in (85), reaching an on-target selectivity in human peripheral blood mononuclear cells (PBMCs) of up to 99% (90).

#### 2.3.2 Application of adenovirus-associated vectors (AAVs) to *in vivo* CAR T-cell generation

Despite their long history in gene therapy, AAVs suffer several limitations for *in vivo* CAR T-cell generation. Their single-stranded DNA genome allows for limited cargo capacity (~4 kb) (91) and for mostly transient transgene delivery, especially in actively proliferating cells such as activated lymphocytes (92). Moreover, due to the lack of an envelope, AAVs need to rely on clathrin-mediated endocytosis for binding and internalization, with the involvement of several capsid and cellular proteins in the process. This adds a layer of complexity to the tweaking of AAV specificity, requiring either the engineering of the capsid proteins or the functionalization of the capsid itself with the binders. Nonetheless, highly selective modification of CD8+ within primary human splenocytes was achieved though DARPin-targeted AAVs (DART-AAVs) (92). Furthermore, AAVs were successfully used for *in vivo* CAR T-cell generation in humanized mouse models (93) The obtained CAR T-cells showed potent, dose-dependent antitumor activity *ex vivo*, and *in vivo* functionality and efficiency comparable to that of conventionally manufactured CAR T-cells (93).

#### 2.3.3 Non-viral technologies for *in vivo* CAR T-cell generation

The effort imbued in the development of SARS-CoV-2 vaccines led to a substantial advancement in non-viral vector technologies. These delivery systems rely on the chemical and physical properties of the payload and carrier combination, rather than on the sophisticated viral machinery. In LNPs, the nucleic acid is encapsulated in a lipid particle through electrostatic interaction, whereas in NCs its negative charges are exploited to complex it with positively charged polymers. They can host DNA or mRNA payloads and better preserve T-cell viability compared to other non-viral delivery technologies, such as electroporation (94). Furthermore, the possibility to be easily functionalized with targeting molecules and their low immunogenicity makes them highly suitable for *in vivo* CAR T-cell generation. In a head-to-head comparison between electroporation and LNPs for the *ex vivo* generation of CAR T-cells via mRNA delivery, LNPs led to prolonged mRNA persistence and CAR surface expression. The obtained CAR T-cells also showed a less exhausted phenotype, likely due to the reduced stress compared to electroporation (94). Both LNPs and NCs proved capable of delivering plasmid DNA and *in vitro*-transcribed RNA cargos to T-cells *in vivo* (95–97), and antibody-conjugated LNPs were specifically targeted toward PECAM-1 (98), CD4 (99) and CD5-positive (96) cell populations.

#### 2.3.4 Current limitations and outstanding issues in *in vivo* CAR T-cell therapy

In general, several factors need to be considered for the clinical translation of *in vivo* strategies. The vast majority of preclinical studies are performed in humanized mouse models, which provide only on-target cells of human origin, making the prediction of the vector biodistribution and of the off-targets in the human body difficult. Likewise, the kinetics of *in vivo*-generated CAR T-cells are necessarily different from their *ex-vivo*-produced counterparts: for the latter, high numbers of effector cells are instantly available upon administration, whereas for the former the vector injection leads to a limited pool of engineered cells, that will have to expand *in vivo* over time. How this difference may affect the behavior and the efficacy of the CAR T-cells in the clinical setting has yet to be elucidated. In all non-integrating technologies, the transgene expression is eventually lost, possibly requiring re-dosing to maintain CAR T-cell levels that ensure long-lasting tumor management. However, the immunogenicity of most vectors for *in vivo* use may be an obstacle for their repeated administration. Neutralizing antibodies have been shown to rise already after the first systemic injection for both LVs and AAVs (9, 100), and pre-existing neutralizing antibodies may be present in a relevant fraction of the patients due to vaccinations or previous infections (101). Lastly, despite the eased manufacturing of *in vivo*-generated CAR T-cells, the availability of sufficient quantities of GMP-grade vectors will be a key factor in the clinical deployment of *in vivo* strategies.

## 3 Engineering strategies

Despite the unprecedented responses obtained with CAR T-cell therapies in hematological malignancies (102–107), these initial successes did not translate into other indications, and results in solid tumors have been especially underwhelming (108, 109). Multiple factors contribute to the poor clinical responses observed in these contexts. The tumor microenvironment (TME) of solid tumors often has immunosuppressive properties and imposes metabolic pressure on the CAR T-cells, thus promoting the formation of dysfunctional effector cells and regulatory T-cells (Treg) (110, 111). As a result, current CAR T-cell products still exhibit poor long-term persistence and exhaustion in solid indications, limiting the duration of responses (112). In addition, a considerable fraction of patients cannot benefit from CAR T-cell therapies due to the poor quality and quantity of T-cells they can provide for autologous CAR T-cell manufacturing. Further engineering is therefore needed to overcome the hurdles encountered so far in the CAR T-cell field (**Table 2**).

### 3.1 Gene editing technologies

The advent and rapid development of gene-editing technologies has given researchers an outstanding toolbox for the engineering of CAR T-cells, allowing for the relatively easy knock-out of undesired genes and knock-in of useful transgenes. Nuclease-based systems, such as zinc-finger nucleases (ZFNs), meganucleases (113), transcription activator-like effector nucleases (TALENs) (114, 115), megaTALs, and clustered regulatory interspaced short palindromic repeat/associated nuclease protein 9 (CRISPR/Cas9) (55, 116)-derived systems all showed potential in CAR T-cell engineering and have been used in multiple clinical applications (117).

All these nuclease-based technologies create genetic modifications by means of the same fundamental principle: they introduce targeted DNA double-strand breaks (DSBs) in the cell genome, which are in turn unfaithfully repaired by the cell’s DSB repair system, thus resulting in the target gene alteration. The two main mechanisms of repair, non-homologous end joining (NHEJ) and homology-directed repair (HDR) (118), are both exploited, to different aims. NHEJ is the predominant repair mechanism in the cell and mediates the direct ligation of the loose DNA ends at DSBs. Its error-prone nature often results in insertions or deletions at the repair site, leading to loss of genetic information or frameshift mutations that eventually knock out the target gene. HDR is less frequent and is mainly active during the late S- and G2- phases of the cell cycle, when DNA replication is completed and the sister chromatids can serve as repair templates. By supplying a template DNA with homology arms to the sequences flanking the DSB, in combination with the targeted nuclease, HDR can be exploited to knock in an exogenous sequence while knocking out the gene of interest. ZFN and TALEN-based technologies rely on protein modules for the target sequence recognition, whereas CRISPR-based systems use an RNA guide for this purpose.

The application of these technologies in CAR T-cell production and the resulting clinical trials have been extensively reviewed elsewhere (15, 119–123). Here we will mainly focus on the current limitations and safety concerns associated with their use in CAR T-cell engineering.

#### 3.1.1 Safety considerations on gene editing technologies

A first risk factor while using nuclease-based genome editing approaches is the possibility of accidentally introducing off-target cleavages. Indeed, Cas9:gRNA complexes can recognize and bind genetic loci with as little as 5-nt of homology with their RNA guide (122), leading to unintended, irreversible off-target genetic alterations. A variety of strategies has been proposed to mitigate such risk, i.e., more sophisticated approaches to gRNA design (124, 125) that rely on a deeper understanding of the parameters leading to off-target binding (126, 127), the use of single-strand nickases along with paired sets of gRNAs (128–130), or the delivery of a pre-formed Cas9:gRNA ribonucleoprotein (RNP) complexes into cells. The latter strategy allows for Cas9 to be active immediately, but the RNP is also quickly degraded once internalized, therefore decreasing the amount of time Cas9 is present for potential off-target cleavage (131, 132).

Field evidences clearly point at the necessity of multiple genetic interventions to overcome the current CAR T-cell limitations, and gene editing technologies have already been explored for the simultaneous targeting of multiple genomic sites in T-cells (133–137). Still, their use for multiplexed gene editing poses relevant biological and technical challenges. Some evidence show that the targeting of Cas9 to different genes simultaneously in human cells could in principle mediate genetic disruptions for each target at efficiencies similar to those achieved by targeting each locus individually (138). However, most often the co-occurrence of all the desired edits within the same cell wanes for high-grade multiplexing (122), posing a serious limit to the scalability and manufacturability of multi-edited products. The competition for a dwindling pool of endonucleases as the number of gRNAs scales up (139) may contribute to this phenomenon, giving raise to unpredictable patterns of genetic modification. To limit cross talk between components, different Cas nucleases have been used in combination, e.g. Cas9 and Cas12, each recognizing a slightly different gRNA structure, thus enabling orthogonal assembly of the ribonucleoprotein complexes (140). The downside of this approach, however, is that a much larger payload needs to be accommodated in the delivery vector, due to the combined presence of the different Cas nucleases.

If the off-target activity and the complexity of multiplexing might be mitigated in the future by advancements in gene-editing technologies, dealing with mutational events and chromosomal rearrangements represents a bigger challenge. Indeed, the biological mechanism behind gene editing itself, which relies on DSBs and on the error-prone DSB-repair machinery, is intrinsically at risk of introducing undesired mutations and chromosomal aberrations. CRISPR/Cas9 cleavage has been linked to gross chromosomal aberrations such as large deletions in early mouse (141, 142) and human embryos (143), as well as in embryonic stem cells and induced pluripotent stem cells (144, 145). More specifically in the CAR T-cell field, CRISPR-based editing of primary human T-cells at the TRAC locus (Ch14q11.2) was shown to lead to chromosome 14 truncation at the cleavage site in 5.3% of the cells (146). Given the acrocentric nature of chromosome 14, this is functionally equivalent to chromosomal loss. Even more strikingly, when the TRAC and TCRB (Ch7q34) loci were co-edited, the percentage of cells with chromosome 14 truncation raised to 9%, along with chromosome 7 truncation at the TCRB locus in 9.9% of the cells (146). This body of evidence is especially relevant for the immune cell therapy field, as TCR disruption is an obliged step for the generation of allogeneic CAR T-cell products. Lastly, the intrinsic *modus operandi* of the repair machinery, that acts stochastically on different alleles in the resolution of DSBs, may lead to intra-allelic mosaicism and, eventually, to batch-scale chimerism even in clonal genetically edited cell products (137, 147, 148).

### 3.2 Evolutions of gene editing technologies

To overcome the risk of off-target cleavages and chromosomal aberrations inherent to classical nuclease-based gene-editing technologies, several innovative strategies are in active development, that do not rely on double-strand breaks and thus offer a potentially safer route to genetic editing. In particular, base editing and prime editing allow the introduction of genetic alterations without the requirement of inducing DSBs.

#### 3.2.1 Base and prime editing

Base editing consists in the sgRNA-directed exchange of single nucleotides mediated by modified forms of Cas9, Cas9 nickases (nCas9), which lack the capacity to cleave DNA, but instead are fused to bacterial deaminases to substitute single nucleotides on just one strand. These point mutations are subsequently resolved during DNA replication, ultimately altering the codon sequence, e.g., to insert premature STOP codons (149, 150). CRISPR-free base editors have also been recently proposed (151). Base editors allow the correction of pathogenic allele variants, holding great promise for the therapy of monogenetic disorders, including sickle cell disease (152), β-hemoglobinopathies (153, 154) and heterozygous familial hypercholesterolemia (155). The first applications in cellular immunotherapy are also emerging, e.g. to engineer fratricide-resistant CAR T-cells by targeting the pan-T lineage antigens CD3 and CD7 (156) and to generate allogeneic CAR T-cells through simultaneous KO of TRAC, CD52, CD7 and PD-1 (157). Compared to CRISPR-edited CAR T-cells, base-edited CAR T-cells showed improved proliferation, lower DNA damage response pathway activation, and no karyotypic abnormalities following multiplexed editing while proving efficacious against T-cell acute lymphoblastic leukemia (T-ALL) both *in vitro* and in preclinical models (157, 158).

Base editing allows for transition mutations (purine-for-purine and pyrimidine-for-pyrimidine substitutions), but not for transversion mutations (swapping purines for pyrimidines and vice versa). Prime editing overcomes this limitation by linking nCas9 to a reverse transcriptase that allows for single base exchanges as well as insertions and deletions of synthetic DNA sequences (159). This technology, however, still suffers from limitations in editing efficiency and in the maximum size of the insert. Efforts are ongoing to improve these aspects, e.g. by designing more efficient guide RNAs for the nCas9 (160) and by building new iterations of the platform with increased payload capacity, such as paired-guide prime editing, which currently allows for insertions of up to ~5 kb (159).

Similarly to standard nuclease-based editors, base and prime editors are at risk of modifying off-target genomic sites. A general strategy to mitigate this risk has consisted in limiting cellular exposure to the editors beyond the duration necessary to achieve the desired on-target editing levels. The delivery of editors and guides as RNP complexes rather than as DNA sequences or the use of small-molecule-controlled editors greatly reduced off-target base editing (161–163), exploiting the faster rate of on-target compared to off-target editing.

#### 3.2.2 Recombinase- and transposase-based gene editing technologies

Targeting the transgene integration to selected loci has been a long-standing goal in gene editing, as it would overcome many of the safety risks associated with insertional mutagenesis. Unfortunately, none of the current clinical-stage engineering technologies fulfills this goal: viral and transposon-based delivery systems do not allow any targeting, whereas nuclease-based, HDR-dependent technologies are limited to actively dividing cells, strongly constraining their applicability. However, novel technologies with the potential of site-directed integration of large payloads are quickly emerging. Phage-derived site-specific recombinases can catalyze the exchange of two dsDNA sequences by recognizing a “landing pad” DNA sequence at the site of insertion. This characteristic has been exploited to mediate the recombination of large cargo sequences in the desired genomic locus pre-installed with the unique landing pads (164). Using this approach, incorporation of very large DNA payloads (>100 kb) with very high efficiency (90%) after selection steps was shown in human iPSCs (4). Site-specific recombinases have remarkable advantages: they do not leave exposed DSBs, there is virtually no upper limit to the size of the cargo, and they are not prone to off-target effects, provided the pre-installed landing pad is inserted precisely on target. Still, their application is rather complex, consisting in multiple steps: the CRISPR-Cas-mediated pre-installment of the landing pads, the delivery of the recombinase-expressing vector and of the DNA payload, the removal of unnecessary auxiliary sequences (e.g. the vector backbones). Each of the steps requires selection and enrichment of the intermediate product cells to cope with the otherwise very low recombination rate (less than 1% in cells containing the landing pad), making this approach not yet scalable nor applicable to most primary cells (4). However, novel evolutions of this technology may overcome these constraints in the near future. Programmable addition via site-specific targeting elements (PASTE) technology, consisting of a Cas9 nickase fused to RT and a serine integrase, can integrate large sequences in human cell lines, primary T cells, and non-dividing primary human hepatocytes with efficiencies between 5% and 60% (165). Lastly, CRISPR-guided transposon systems (CAST), combining the site-specificity of a catalytically inactive Cas with the ability of the transposon machinery to integrate the cargo in the host genome, have been used to deliver payloads of up to 10 kb (166–168), although their applications has been so far limited to gene editing in bacteria.

Although these novel approaches do not yet show efficiencies required for clinical scalability and manufacturing and have not been sufficiently tested in primary cells, the extremely rapid evolution of the field may soon bring them closer to be applied in clinical settings.

### 3.3 Non-gene editing technologies

All gene editing technologies directly intervene on the DNA sequence, with two unavoidable consequences: the risk of undesirable genomic mutations and the irreversibility of the modifications. On the other hand, non-gene editing technologies do not directly alter the genetic information of the host cell, negating the risk of detrimental genetic alterations and opening the possibility of modulating the intensity of their effect, rather than being an on/off system.

#### 3.3.1 CRISPR activation (CRISPRa) and CRISPR inhibition/interference (CRISPRi)

Branching from the standard CRISPR/Cas9 gene editing approach, catalytically disabled Cas9 (dCas9) have been fused to transcriptional modulators. The dCas9 lacks its catalytic activity, but retains its sequence-specific binding ability, thus allowing the transcriptional modulators to alter the target gene expression at the epigenetic or transcriptional level, without the need of DSBs (169). This approach can be used to enhance or repress the transcription of the target gene. CRISPR activation (CRISPRa) exploits effectors such as VP64, VPR, Suntag, p300, and Synergistic activation mediator (SAM) to induce epigenetic changes, e.g. histone acetylation, that lead to enhanced gene expression (169, 170). CRISPR inhibition/interference (CRISPRi) uses epigenetic regulators of DNA methylation, histone acetylation, or histone methylation, e.g. Krüppel-associated box (KRAB), DNMT3A, and HDAC, to downregulate gene expression (169, 170). Recently, a tool for programmable epigenetic memory based on DNA methylation, named CRISPRoff, has also been described, that can make such gene inhibition heritable (171). CRISPRa and CRISPRi have mainly been used in functional genomics screenings thus far. A genome-wide CRISPRi/CRISPRa screening in primary human T-cells was employed to identify therapeutically relevant T cell states which may prove useful in the design of novel T cell-based immunotherapies (172). Likewise, a CRISPRa gain-of-function screening in murine CAR T-cells highlighted proline metabolism as a driver of CAR T-cell fitness and function (173). Still, the first applications in cell engineering are already emerging, notably also in contexts such as iPSCs (170) and primary T-cells (172, 174). Specifically, multiplex gene modulation of up to four genes (*ITGA3*, *THY1*, *IL3RA*, and *NGFR*) was achieved in human CD34+ hematopoietic stem and progenitor cells (HSPCs) and human CD3+ T-cells, without any discernible detrimental effects on the HSPCs capacity to engraft long-term in immunodeficient mice and on T-cell expansion (174). CRISPRa and CRISPRi have also been exploited to tweak primary T-cell responses, e.g. cytokine secretion, by impinging on their regulatory pathways (172). Interestingly, both the magnitude and the duration of the CRIPSRa/CRISPRi effect could be tuned by careful sgRNA selection, and further tuning may also be possible by adapting other sgRNA properties, such as the poly(A) tail length, codon usage, and incorporation of modified nucleotides (174). In terms of safety, several studies have found CRISPRa and CRISPRi to be highly specific (175–177). In contrast to gene editing with nuclease-active Cas9, the combination with epigenetic effectors makes CRISPRa and CRISPRi only active within a short distance from the targeted transcription start sites. In addition, the extent of the potential adverse effects is mitigated by the reversible nature of the modifications and can be further reduced by temporally restraining the activity of the editors via their transient delivery as RNP complexes.

#### 3.3.2 mRNA-targeting approaches: CRISPR/Cas13

A radically different approach for non-gene editing technologies consists in targeting the mRNA of the gene of interest, rather than its genomic sequence, thus inducing defined cellular phenotypes without introducing genomic alterations. In this light, the recent discovery of CRISPR/Cas13, a Cas-based system that uses CRISPR RNA guides to target RNA, spurred considerable interest (178, 179). This Cas allows for direct transcriptome engineering via RNA editing and KD, without the requirement for permanent genetic manipulations (180–182). In early reports CRISPR/Cas13 mediated potent and specific target RNA downregulation in eukaryotic cells, outperforming CRISPRi, apparently with minimal off-target transcriptome changes (178, 180, 183). The system has since been adapted for use in a variety of contexts, from yeast (184) to Drosophila (185) to mice (186–189), and has been applied to neutralize viral infections in animal models (190, 191). However, the high specificity of CRISPR/Cas13 in eukaryotic cells observed in early studies represents a paradox. Indeed, due to its molecular architecture, with the catalytic site located on the outside of the protein, facing away from the guide RNA-target RNA complex, Cas13 is prone to indiscriminately cleave any bystander RNA. This effect, termed collateral cleavage activity, has been observed both *in vitro* and in bacteria (192). Coherently, several recent publications report Cas13-mediated toxicity and collateral RNA cleavage in eukaryotes (reviewed in (192)), although the reason for the discrepancies with earlier studies is still matter of investigation.

#### 3.3.3 mRNA-targeting approaches: RNA interference (RNAi)

A more established approach for transcriptional regulation, RNA interference (RNAi), has also been tweaked and adapted in the last few years for the engineering of CAR T-cells, with interesting results. RNAi is a bundle of technologies, namely small interfering RNAs (siRNAs), short hairpin RNAs (shRNAs), and microRNAs (miRNAs), all based on small non-coding RNAs that regulate gene expression post-transcriptionally. They do not require a direct intervention on the target’s genomic sequence and thus they do not incur the risk of causing genomic alterations.

siRNAs are delivered in their mature, functionally active form, with no possibility to self-replenish the initial siRNA pool. Hence, siRNAs are not suitable for the development of engineered immune cells. Indeed, the rapid dilution of the siRNAs in the fast-dividing activated T cells makes this approach only applicable to obtain transient biological effect. On the other hand, shRNAs and miRNAs are continuously transcribed as precursors in the recipient cells, ensuring the long-term gene downregulation required for effective CAR T-cell engineering. shRNAs and miRNAs share the same molecular machinery for their maturation: following transcription, the immature hairpin structure is processed by RNase III enzymes (first Drosha, then DICER in the case of miRNAs, only DICER in the case of shRNAs), leading to the formation of a mature RNA duplex, which is in turn incorporated into the RNA-induced silencing complex (RISC). The accessory passenger RNA strand is then released and the RISC-guide strand riboprotein mediates target mRNA recognition and downregulation (193–195). Two important differences exist in the biogenesis of shRNAs and miRNAs. First, shRNAs are transcribed by RNA polymerase III, whereas miRNAs are driven by RNA polymerase II. Hence, miRNAs are usually expressed at lower, more tolerable levels than shRNAs and can be easily embedded in polycistronic transcriptional units that facilitate CAR T-cell engineering (196). Second, in addition to the very high expression levels, shRNA bypass Drosha during their maturation process, possibly overloading the cytoplasm with double-stranded RNA which may obstruct the natural miRNA pathway and thus lead to toxicity (197, 198). Synthetic miRNAs, in which the guide sequence has been swapped for an shRNA-based one directed against the gene of interest, can overcome this issue, as they still closely exploit the natural miRNA pathway (199), standing out as the most valid RNAi technology for immune cell engineering. Clinical validation of the miRNA-based RNAi approach in CAR T-cells has been obtained in two phase I clinical trials (NCT04613557, NCT03466320). These studies show both the functional efficiency and the high safety profile of the technology.

Beyond the aforementioned examples, RNAi has been used to engineer various features of CAR T-cells, with promising results in preclinical models, and some of these strategies are currently under evaluation in clinical trials (NCT06051695, NCT05617755, NCT06245915, NCT04649112, NCT04825496, NCT05028933). Notable applications are the downregulation of IFNγ or GM-CSF to reduce the risk of cytokine release syndrome (200, 201), the targeting of immune checkpoints to inhibit CAR T-cell exhaustion and enhance functionality (136, 202), the silencing of factors, such as adenosine 2A receptor (A2aR), to improve resistance to the immunosuppressive tumor microenvironment in the context of solid tumors (203), and the simultaneous knock-down of TCR and HLA class I for the generation of allogeneic CAR T-cells (196). These applications elucidate one of the greatest advantages of RNAi over other engineering technologies, i.e. the possibility to modulate the expression of the target gene to levels compatible with its biology, reaching functionally relevant levels of inhibition without the detrimental effects that a full KO may cause.

Another valuable characteristic of miRNAs is their frequent co-occurrence within the same transcriptional unit within the genome. About 50% of conserved vertebrate miRNAs are organized in clusters (194, 204), making them an ideal engineering strategy for multiplex applications. miRNA systems for multiple gene targeting have already been used successfully against HIV-1 and HCV (205–207), although their clinical applicability in immune cell therapy has been hampered by the relatively weak knock-down efficiencies obtained thus far (208). More recently, however, miRNA-based multiplex platforms able to effectively target up to four genes have been proposed for the engineering of CAR T-cells (196, 209) and optimized to reach efficiencies compatible with the use in clinical products (196). Overall, multiplex RNAi technologies allow for an easy, safe, efficient, and tunable modulation of several genes simultaneously.

## 4 Perspective: engineering a new generation of safer and more effective CAR T-cells

The CAR T-cell revolution gave hope to patients who did not have a valid therapeutic option. Still, for many indications, and especially for solid tumors, the current CAR T-cell design is not sufficient to succeed in these challenges, thus requiring further modifications. In the vast majority of novel CAR T-cell products, these are achieved via gene editing, mainly with CRISPR/Cas9. The mechanism of action of gene editing technologies is intrinsically at risk of creating undesired mutations and chromosomal aberrations in the modified cells, and preclinical data convincingly confirm that this is not a remote eventuality (141–145). While clinical trials are not yet conclusive regarding genotoxicity due to gene editing, several factors need to be considered. Indeed, the CRISPR technology is still relatively young and only recently moved into clinics, with the consequence that the amount of clinical data and the follow-up time do not yet allow for an in-depth estimate of its safety profile in patients. Moreover, in many gene-edited products, CRISPR technology is used for the generation of allogeneic CAR T-cells, whose persistence is still limited compared to their autologous counterparts. The rise of harmful CAR T-cell clones may therefore be counteracted by their fast *in vivo* clearance.

While the genotoxicity risk is still hypothetical in the case of gene editing, secondary tumors correlated to the transgene delivery system have been observed, both for viral vectors and for transposons. As these two strategies cover the manufacturing of the vast majority of CAR T-cells at preclinical and clinical level, a systematic, albeit low, risk of genotoxicity is virtually present in all CAR T-cell products. Gene editing adds a further layer of complexity to the picture, as future products will be burdened by the combined hazard coming from both the gene delivery system and the engineering technology. Mitigation measures will therefore become crucial for the future of immune cell therapies: reinforced long-term surveillance for secondary tumors will likely be a requirement, while alternative, safer technologies, such as base and prime editing, will need to be promptly ameliorated for broader clinical applicability. Overall, the investments aimed at improving the safety of CAR T-cell products will be as important as the ones concerning efficacy and persistence. Currently, the best compromise between manufacturability, efficacy and safety may be represented by the combination of lentiviral-based vectors and more established non-gene editing technologies such as RNAi. Non-gene edited technologies also present the added value of allowing the modulation of gene expression, a characteristic of great relevance when targeting genes that impinge on delicate biological equilibriums, such as activation/exhaustion and cell metabolism, and whose complete ablation would be detrimental.

*In vivo* technologies look especially promising, as their application can be envisioned beyond the mere deployment of the CAR components. Both viral and non-viral vectors are being actively tested for *in vivo* CRISPR delivery and editing (210–212), for oncological as well as for gene therapy applications. Even more interestingly, *in vivo* targeting can be envisioned for the modification of other components of TME (213), such as macrophages, regulatory cells and the cancer cells themselves, thus manipulating the cytokine, immune checkpoints and metabolite milieu. Several areas of intervention could be envisioned (reviewed in (3)). CAR T-cell penetration into the tumor could be enhanced by acting on the tumor vasculature or on cancer associated fibroblasts (CAFs), or locally expressing matrix-degrading enzymes. CAR T-cell functionality could be better preserved by acting on immunosuppressive immune cells in the TME (T_regs_, tumor-associated fibroblasts, myeloid-derived suppressor cells), on immunosuppressive soluble factors (such as TGF-β and IL-4), or on inhibitory receptors and ligands expressed by tumor or stromal cells (such as immune checkpoint ligands). Such a combinatorial approach may create a more permissive environment for CAR T-cells to operate, granting unprecedented possibilities of success for unmet medical needs. An interesting novel way by which the safety of CAR T-cell therapy could be enhanced involves the use of CAR T-cell-derived Extracellular Vesicles (EVs). Expression of the CAR on the EV surface renders the EVs responsive to the CAR antigen, and incorporation of perforin and granzyme B in the EV cargo leads to lysis of the target cell. As EVs do not proliferate and have a limited life span, they hold several advantages in terms of safety. First, they do not pose the risk of secondary cancers development. Second, the potential CAR T-cell related toxicity is significantly reduced, as the lack of proliferation means a reduction in both CRS and ICANS (214). Third, CAR T-cell derived EVs have a relative low immunogenicity in heterologous infusions, and may easily cross the tumor barrier, as observed by tumor-derived EVs in both body fluids (215, 216) and tumors that are characterized by strong fibrotic reaction (217). Preclinical studies using anti-EGFR and HER-2 CAR T-cell-derived EVs demonstrated no toxicity combined with a high efficacy in HER+ and EGFR+ xenograft mouse models. Moreover, CAR T-cell-derived EVs were insensitive to PD-L1 immunosuppression, suggesting an interesting approach to avoid immune suppression induced by immune checkpoints (214). A similar study conducted with mesothelin-targeted CAR-T derived EVs showed comparable results with no signs of CRS combined with high efficacy (218). However, despite their favorable safety profile, the potency of CAR T-cell-derived EVs has yet to be assessed properly in the clinical setting, as well as their clinical-grade manufacturability.

Following the brilliant results obtained by anti CD19 CAR T-cells in autoimmune diseases (39, 42, 219), the immune cell therapy field is rapidly expanding to non-oncological indications. This welcome advancement, however, brings forward outstanding safety considerations. Indeed, if the current rate of CAR T-cell-derived secondary malignancies may still be within a favorable risk-to-benefit ratio for oncological patients, it would be unacceptable for individuals that have alternative treatments and do not suffer from life-threatening diseases. In this light, manufacturing and engineering technologies that showed limitations in cancer therapy could represent a valid, equally effective but safer alternative in other indications. The low persistence of current allogeneic CAR T-cell therapies does not ensure proper long-term tumor management but may turn into a favorable characteristic to minimize the risk of secondary lymphomas in autoimmune patients. Likewise, manufacturing strategies based on transient CAR expression and *in vivo*-generated CAR T-cells may express all their potential in indications other than oncology. A longer follow-up on a wider cohort of patients will indicate whether an acute treatment with shortly persisting CAR T-cells will be sufficient to lead to durable responses against autoimmune diseases.

For the next future, it is therefore conceivable that different indications will benefit of different CAR T-cell designs, manufacturing, and engineering strategies. Autologous, allogeneic, and *in vivo*-generated CAR T-cells will likely coexist in the immune cell therapy toolbox and their employment against different diseases will be tailored on their characteristics and strengths.

## Author contributions

MR: Writing – original draft, Writing – review & editing. EB: Writing – original draft, Writing – review & editing.

## References

[1] Rotte A Frigault MJ Ansari A Gliner B Heery C Shah B. Dose–response correlation for CAR-T cells: a systematic review of clinical studies. J Immunother Cancer. (2022) 10:e005678. doi: 10.1136/jitc-2022-005678 36549782 PMC9791395

[2] Cappell KM Kochenderfer JN. Long-term outcomes following CAR T cell therapy: what we know so far. Nat Rev Clin Oncol. (2023) 20:359–71. doi: 10.1038/s41571-023-00754-1 PMC10100620 37055515

[3] Rodriguez-Garcia A Palazon A Noguera-Ortega E Powell DJ Guedan S. CAR-T cells hit the tumor microenvironment: strategies to overcome tumor escape. Front Immunol. (2020) 11:1109/full. doi: 10.3389/fimmu.2020.01109/full 32625204 PMC7311654

[4] Fichter KM Setayesh T Malik P. Strategies for precise gene edits in mammalian cells. Mol Ther - Nucleic Acids. (2023) 32:536–52. doi: 10.1016/j.omtn.2023.04.012 PMC10192336 37215153

[5] Morgan R Boyerinas B. Genetic modification of T cells. Biomedicines. (2016) 4:9. doi: 10.3390/biomedicines4020009 28536376 PMC5344249

[6] Labbé RP Vessillier S Rafiq QA. Lentiviral vectors for T cell engineering: clinical applications, bioprocessing and future perspectives. Viruses. (2021) 13:1528. doi: 10.3390/v13081528 34452392 PMC8402758

[7] Ayala Ceja M Khericha M Harris CM Puig-Saus C Chen YY. CAR-T cell manufacturing: Major process parameters and next-generation strategies. J Exp Med. (2024) 221:1–14. doi: 10.1084/jem.20230903 PMC10791545 38226974

[8] Lundstrom K. Viral vectors in gene therapy: where do we stand in 2023? Viruses. (2023) 15:698. doi: 10.3390/v15030698 36992407 PMC10059137

[9] Abordo-Adesida E Follenzi A Barcia C Sciascia S Castro MG Naldini L. Stability of lentiviral vector-mediated transgene expression in the brain in the presence of systemic antivector immune responses. Hum Gene Ther. (2005) 16:741–51. doi: 10.1089/hum.2005.16.741 PMC2676203 15960605

[10] Baekelandt V Eggermont K Michiels M Nuttin B Debyser Z. Optimized lentiviral vector production and purification procedure prevents immune response after transduction of mouse brain. Gene Ther. (2003) 10:1933–40. doi: 10.1038/sj.gt.3302094 14528317

[11] Marcucci KT Jadlowsky JK Hwang WT Suhoski-Davis M Gonzalez VE Kulikovskaya I. Retroviral and lentiviral safety analysis of gene-modified T cell products and infused HIV and oncology patients. Mol Ther. (2018) 26:269–79. doi: 10.1016/j.ymthe.2017.10.012 PMC5763152 29203150

[12] Scholler J Brady TL Binder-Scholl G Hwang WT Plesa G Hege KM. Decade-long safety and function of retroviral-modified chimeric antigen receptor T cells. Sci Transl Med. (2012) 4:132–53. doi: 10.1126/scitranslmed.3003761 PMC4368443 22553251

[13] Cesana D Ranzani M Volpin M Bartholomae C Duros C Artus A. Uncovering and dissecting the genotoxicity of self-inactivating lentiviral vectors in vivo. Mol Ther. (2014) 22:774–85. doi: 10.1038/mt.2014.3 PMC3982501 24441399

[14] Milone MC O’Doherty U. Clinical use of lentiviral vectors. Leukemia. (2018) 32:1529–41. doi: 10.1038/s41375-018-0106-0 PMC6035154 29654266

[15] Irving M Lanitis E Migliorini D Ivics Z Guedan S. Choosing the right tool for genetic engineering: clinical lessons from chimeric antigen receptor-T cells. Hum Gene Ther. (2021) 32:1044–58. doi: 10.1089/hum.2021.173 PMC8697565 34662233

[16] Center for Biologics Evaluation and Research. Testing of retroviral vector-based human gene therapy products for replication competent retrovirus during product manufacture and patient follow-up. U.S. Food & Drug Administration (2020). Available at: https://www.fda.gov/regulatory-information/search-fda-guidance-documents/testing-retroviral-vector-based-human-gene-therapy-products-replication-competent-retrovirus-during.

[17] Ruella M Xu J Barrett DM Fraietta JA Reich TJ Ambrose DE. Induction of resistance to chimeric antigen receptor T cell therapy by transduction of a single leukemic B cell. Nat Med. (2018) 24:1499–503. doi: 10.1038/s41591-018-0201-9 PMC6511988 30275568

[18] Frank AM Braun AH Scheib L Agarwal S Schneider IC Fusil F. Combining T-cell-specific activation and in *vivo* gene delivery through CD3-targeted lentiviral vectors. Blood Adv. (2020) 4:5702–15. doi: 10.1182/bloodadvances.2020002229 PMC7686896 33216892

[19] Desfarges S Ciuffi A. Viral Integration and Consequences on Host Gene Expression. In: Witzany G, editor. Viruses: Essential Agents of Life. Springer Netherlands, Dordrecht (2012). p. 147–75. doi: 10.1007/978-94-007-4899-6_7

[20] Howe SJ Mansour MR Schwarzwaelder K Bartholomae C Hubank M Kempski H. Insertional mutagenesis combined with acquired somatic mutations causes leukemogenesis following gene therapy of SCID-X1 patients. J Clin Invest. (2008) 118:3143–50. doi: 10.1172/JCI35798 PMC2496964 18688286

[21] Timms RT Tchasovnikarova IA Lehner PJ. Differential viral accessibility (DIVA) identifies alterations in chromatin architecture through large-scale mapping of lentiviral integration sites. Nat Protoc. (2019) 14:153–70. doi: 10.1038/s41596-018-0087-5 30518911

[22] Wells DW Guo S Shao W Bale MJ Coffin JM Hughes SH. An analytical pipeline for identifying and mapping the integration sites of HIV and other retroviruses. BMC Genomics. (2020) 21:216. doi: 10.1186/s12864-020-6647-4 32151239 PMC7063773

[23] Huang KB Guo SJ Li YH Zhang XK Chen D Spiess PE. Genome-wide profiling reveals HPV integration pattern and activated carcinogenic pathways in penile squamous cell carcinoma. Cancers. (2021) 13:6104. doi: 10.3390/cancers13236104 34885212 PMC8657281

[24] Ferber MJ Thorland EC Brink AA Rapp AK Phillips LA McGovern R. Preferential integration of human papillomavirus type 18 near the c-myc locus in cervical carcinoma. Oncogene. (2003) 22:7233–42. doi: 10.1038/sj.onc.1207006 14562053

[25] De Ravin SS Su L Theobald N Choi U Macpherson JL Poidinger M. Enhancers are major targets for murine leukemia virus vector integration. Beemon KL editor J Virol. (2014) 88:4504–13. doi: 10.1128/JVI.00011-14 PMC3993722 24501411

[26] Kraus I Driesch C Vinokurova S Hovig E Schneider A von Knebel Doeberitz M. The majority of viral-cellular fusion transcripts in cervical carcinomas cotranscribe cellular sequences of known or predicted genes. Cancer Res. (2008) 68:2514–22. doi: 10.1158/0008-5472.CAN-07-2776 18381461

[27] Cavazza A Moiani A Mavilio F. Mechanisms of retroviral integration and mutagenesis. Hum Gene Ther. (2013) 24:119–31. doi: 10.1089/hum.2012.203 23330935

[28] Beard BC Dickerson D Beebe K Gooch C Fletcher J Okbinoglu T. Comparison of HIV-derived lentiviral and MLV-based gammaretroviral vector integration sites in primate repopulating cells. Mol Ther. (2007) 15:1356–65. doi: 10.1038/sj.mt.6300159 17440443

[29] Shao L Shi R Zhao Y Liu H Lu A Ma J. Genome-wide profiling of retroviral DNA integration and its effect on clinical pre-infusion CAR T-cell products. J Transl Med. (2022) 20:514. doi: 10.1186/s12967-022-03729-5 36348415 PMC9644589

[30] Hacein-Bey-Abina S von Kalle C Schmidt M Le Deist F Wulffraat N McIntyre E. A serious adverse event after successful gene therapy for X-linked severe combined immunodeficiency. N Engl J Med. (2003) 348:255–6. doi: 10.1056/NEJM200301163480314 12529469

[31] Ott MG Schmidt M Schwarzwaelder K Stein S Siler U Koehl U. Correction of X-linked chronic granulomatous disease by gene therapy, augmented by insertional activation of MDS1-EVI1, PRDM16 or SETBP1. Nat Med. (2006) 12:401–9. doi: 10.1038/nm1393 16582916

[32] Zychlinski D Schambach A Modlich U Maetzig T Meyer J Grassman E. Physiological promoters reduce the genotoxic risk of integrating gene vectors. Mol Ther. (2008) 16:718–25. doi: 10.1038/mt.2008.5 18334985

[33] Emmons TR Giridharan T Singel KL Khan ANH Ricciuti J Howard K. Mechanisms driving neutrophil-induced T-cell immunoparalysis in ovarian cancer. Cancer Immunol Res. (2021) 9:790–810. doi: 10.1158/2326-6066.CIR-20-0922 33990375 PMC8287091

[34] Singel KL Emmons TR Khan ANH Mayor PC Shen S Wong JT. Mature neutrophils suppress T cell immunity in ovarian cancer microenvironment. JCI Insight. (2019) 4:e122311. doi: 10.1172/jci.insight.122311 30730851 PMC6483507

[35] Fraietta JA Nobles CL Sammons MA Lundh S Carty SA Reich TJ. Disruption of TET2 promotes the therapeutic efficacy of CD19-targeted T cells. Nature. (2018) 558:307–12. doi: 10.1038/s41586-018-0178-z PMC6320248 29849141

[36] Shah NN Johnson BD Schneider D Zhu F Szabo A Keever-Taylor CA. Bispecific anti-CD20, anti-CD19 CAR T cells for relapsed B cell Malignancies: a phase 1 dose escalation and expansion trial. Nat Med. (2020) 26:1569–75. doi: 10.1038/s41591-020-1081-3 33020647

[37] Verdun N Marks P. Secondary cancers after chimeric antigen receptor T-cell therapy. N Engl J Med. (2024) 390:584–6. doi: 10.1056/NEJMp2400209 38265704

[38] Mackensen A Müller F Mougiakakos D Böltz S Wilhelm A Aigner M. Anti-CD19 CAR T cell therapy for refractory systemic lupus erythematosus. Nat Med. (2022) 28:2124–32. doi: 10.1038/s41591-022-02017-5 36109639

[39] Müller F Taubmann J Bucci L Wilhelm A Bergmann C Völkl S. CD19 CAR T-cell therapy in autoimmune disease — A case series with follow-up. N Engl J Med. (2024) 390:687–700. doi: 10.1056/NEJMoa2308917 38381673

[40] Pecher AC Hensen L Klein R Schairer R Lutz K Atar D. CD19-targeting CAR T cells for myositis and interstitial lung disease associated with antisynthetase syndrome. JAMA. (2023) 329:2154. doi: 10.1001/jama.2023.8753 37367976 PMC10300719

[41] Morte-Romea E Pesini C Pellejero-Sagastizábal G Letona-Giménez S Martínez-Lostao L Aranda SL. CAR Immunotherapy for the treatment of infectious diseases: a systematic review. Front Immunol. (2024) 15:1289303/full. doi: 10.3389/fimmu.2024.1289303/full 38352878 PMC10861799

[42] Chasov V Zmievskaya E Ganeeva I Gilyazova E Davletshin D Khaliulin M. Immunotherapy strategy for systemic autoimmune diseases: betting on CAR-T cells and antibodies. Antibodies. (2024) 13:10. doi: 10.3390/antib13010010 38390871 PMC10885098

[43] Center for Biologics Evaluation and Research. Considerations for the development of chimeric antigen receptor (CAR) T cell products. U.S. Food & Drug Administration (2022). Available at: https://www.fda.gov/regulatory-information/search-fda-guidance-documents/considerations-development-chimeric-antigen-receptor-car-t-cell-products.

[44] Young RM Engel NW Uslu U Wellhausen N June CH. Next-generation CAR T-cell therapies. Cancer Discovery. (2022) 12:1625–33. doi: 10.1158/2159-8290.CD-21-1683 PMC9262817 35417527

[45] McClintock B. The origin and behavior of mutable loci in maize. Proc Natl Acad Sci. (1950) 36:344–55. doi: 10.1073/pnas.36.6.344 PMC1063197 15430309

[46] Ivics Z Li MA Mátés L Boeke JD Nagy A Bradley A. Transposon-mediated genome manipulation in vertebrates. Nat Methods. (2009) 6:415–22. doi: 10.1038/nmeth.1332 PMC2867038 19478801

[47] Ivics Z Hackett PB Plasterk RH Izsvák Z. Molecular reconstruction of sleeping beauty, a tc1-like transposon from fish, and its transposition in human cells. Cell. (1997) 91:501–10. doi: 10.1016/S0092-8674(00)80436-5 9390559

[48] Fraser MJ Smith GE Summers MD. Acquisition of Host Cell DNA Sequences by Baculoviruses: Relationship Between Host DNA Insertions and FP Mutants of *Autographa californica* and *Galleria mellonella* Nuclear Polyhedrosis Viruses. J Virol. (1983) 47:287–300. doi: 10.1128/jvi.47.2.287-300.1983 16789244 PMC255260

[49] Rostovskaya M Fu J Obst M Baer I Weidlich S Wang H. Transposon-mediated BAC transgenesis in human ES cells. Nucleic Acids Res. (2012) 40:e150–0. doi: 10.1093/nar/gks643 PMC3479164 22753106

[50] Magnani CF Gaipa G Lussana F Belotti D Gritti G Napolitano S. Sleeping Beauty–engineered CAR T cells achieve antileukemic activity without severe toxicities. J Clin Invest. (2020) 130:6021–33. doi: 10.1172/JCI138473 PMC7598053 32780725

[51] Magnani CF Mezzanotte C Cappuzzello C Bardini M Tettamanti S Fazio G. Preclinical efficacy and safety of CD19CAR cytokine-induced killer cells transfected with sleeping beauty transposon for the treatment of acute lymphoblastic leukemia. Hum Gene Ther. (2018) 29:602–13. doi: 10.1089/hum.2017.207 29641322

[52] Fischer SEJ van Luenen HGAM Plasterk RHA. Cis requirements for transposition of Tc1-like transposons in C. elegans. Mol Gen Genet MGG. (1999) 262:268–74. doi: 10.1007/PL00008641 10517322

[53] Izsvák Z Ivics Z Plasterk RH. Sleeping Beauty, a wide host-range transposon vector for genetic transformation in vertebrates 1 1Edited by J. Karn J Mol Biol. (2000) 302:93–102. doi: 10.1006/jmbi.2000.4047 10964563

[54] Oh SA Senger K Madireddi S Akhmetzyanova I Ishizuka IE Tarighat S. High-efficiency nonviral CRISPR/Cas9-mediated gene editing of human T cells using plasmid donor DNA. J Exp Med. (2022) 219:e20211530. doi: 10.1084/jem.20211530 35452075 PMC9040063

[55] Roth TL Puig-Saus C Yu R Shifrut E Carnevale J Li PJ. Reprogramming human T cell function and specificity with non-viral genome targeting. Nature. (2018) 559:405–9. doi: 10.1038/s41586-018-0326-5 PMC6239417 29995861

[56] Balke-Want H Keerthi V Cadinanos-Garai A Fowler C Gkitsas N Brown AK. Non-viral chimeric antigen receptor (CAR) T cells going viral. Immuno-Oncol Technol. (2023) 18:100375. doi: 10.1016/j.iotech.2023.100375 PMC10139995 37124148

[57] Monjezi R Miskey C Gogishvili T Schleef M Schmeer M Einsele H. Enhanced CAR T-cell engineering using non-viral Sleeping Beauty transposition from minicircle vectors. Leukemia. (2017) 31:186–94. doi: 10.1038/leu.2016.180 27491640

[58] Querques I Mades A Zuliani C Miskey C Alb M Grueso E. A highly soluble Sleeping Beauty transposase improves control of gene insertion. Nat Biotechnol. (2019) 37:1502–12. doi: 10.1038/s41587-019-0291-z PMC6894935 31685959

[59] Mátés L Chuah MKL Belay E Jerchow B Manoj N Acosta-Sanchez A. Molecular evolution of a novel hyperactive Sleeping Beauty transposase enables robust stable gene transfer in vertebrates. Nat Genet. (2009) 41:753–61. doi: 10.1038/ng.343 19412179

[60] Jin Z Maiti S Huls H Singh H Olivares S Mátés L. The hyperactive Sleeping Beauty transposase SB100X improves the genetic modification of T cells to express a chimeric antigen receptor. Gene Ther. (2011) 18:849–56. doi: 10.1038/gt.2011.40 PMC4083583 21451576

[61] Voigt F Wiedemann L Zuliani C Querques I Sebe A Mátés L. Sleeping Beauty transposase structure allows rational design of hyperactive variants for genetic engineering. Nat Commun. (2016) 7:11126. doi: 10.1038/ncomms11126 27025571 PMC4820933

[62] Yusa K Zhou L Li MA Bradley A Craig NL. A hyperactive PiggyBac transposase for mammalian applications. Proc Natl Acad Sci. (2011) 108:1531–6. doi: 10.1073/pnas.1008322108 PMC3029773 21205896

[63] Amberger M Ivics Z. Latest Advances for the Sleeping Beauty Transposon System: 23 Years of Insomnia but Prettier than Ever: Refinement and Recent Innovations of the Sleeping Beauty Transposon System Enabling Novel, Nonviral Genetic Engineering Applications. BioEssays. (2020) 42:e2000136. doi: 10.1002/bies.202000136 32939778

[64] Moretti A Ponzo M Nicolette CA Tcherepanova IY Biondi A Magnani CF. The past, present, and future of non-viral CAR T cells. Front Immunol. (2022) 13:867013/full. doi: 10.3389/fimmu.2022.867013/full 35757746 PMC9218214

[65] Bishop DC Clancy LE Simms R Burgess J Mathew G Moezzi L. Development of CAR T-cell lymphoma in 2 of 10 patients effectively treated with piggyBac-modified CD19 CAR T cells. Blood. (2021) 138:1504–9. doi: 10.1182/blood.2021010813 34010392

[66] Micklethwaite KP Gowrishankar K Gloss BS Li Z Street JA Moezzi L. Investigation of product-derived lymphoma following infusion of *piggyBac* -modified CD19 chimeric antigen receptor T cells. Blood. (2021) 138:1391–405. doi: 10.1182/blood.2021010858 PMC8532197 33974080

[67] Park J Daniels J Wartewig T Ringbloom KG Martinez-Escala ME Choi S. Integrated genomic analyses of cutaneous T-cell lymphomas reveal the molecular bases for disease heterogeneity. Blood. (2021) 138:1225–36. doi: 10.1182/blood.2020009655 PMC8499046 34115827

[68] Daniels J Choi J. BACH2 is a putative T-cell lymphoma tumor suppressor that may play a role in product-derived CAR T-cell lymphomas. Blood. (2021) 138:2731–3. doi: 10.1182/blood.2021012641 PMC8703361 34499707

[69] Gogol-Döring A Ammar I Gupta S Bunse M Miskey C Chen W. Genome-wide profiling reveals remarkable parallels between insertion site selection properties of the MLV retrovirus and the piggyBac transposon in primary human CD4+ T cells. Mol Ther. (2016) 24:592–606. doi: 10.1038/mt.2016.11 26755332 PMC4786924

[70] Sandoval-Villegas N Nurieva W Amberger M Ivics Z. Contemporary Transposon Tools: A Review and Guide through Mechanisms and Applications of Sleeping Beauty, piggyBac and Tol2 for Genome Engineering. Int J Mol Sci. (2021) 22:5084. doi: 10.3390/ijms22105084 34064900 PMC8151067

[71] de Jong J Akhtar W Badhai J Rust AG Rad R Hilkens J. Chromatin landscapes of retroviral and transposon integration profiles. PloS Genet. (2014) 10:e1004250. doi: 10.1371/journal.pgen.1004250 24721906 PMC3983033

[72] Vigdal TJ Kaufman CD Izsvák Z Voytas DF Ivics Z. Common physical properties of DNA affecting target site selection of sleeping beauty and other tc1/mariner transposable elements. J Mol Biol. (2002) 323:441–52. doi: 10.1016/S0022-2836(02)00991-9 12381300

[73] Henssen AG Henaff E Jiang E Eisenberg AR Carson JR Villasante CM. Genomic DNA transposition induced by human PGBD5. eLife. (2015) 4:e10565. doi: 10.7554/eLife.10565 26406119 PMC4625184

[74] Helou L Beauclair L Dardente H Piégu B Tsakou-Ngouafo L Lecomte T. The piggyBac-derived protein 5 (PGBD5) transposes both the closely and the distantly related piggyBac-like elements Tcr-pble and Ifp2. J Mol Biol. (2021) 433:166839. doi: 10.1016/j.jmb.2021.166839 33539889 PMC8404143

[75] Henssen AG Koche R Zhuang J Jiang E Reed C Eisenberg A. PGBD5 promotes site-specific oncogenic mutations in human tumors. Nat Genet. (2017) 49:1005–14. doi: 10.1038/ng.3866 PMC5489359 28504702

[76] Beckermann TM Luo W Wilson CM Veach RA Wilson MH. Cognate restriction of transposition by *piggyBac-* like proteins. Nucleic Acids Res. (2021) 49:8135–44. doi: 10.1093/nar/gkab578 PMC8373079 34232995

[77] Bozza M De Roia A Correia MP Berger A Tuch A Schmidt A. A nonviral, nonintegrating DNA nanovector platform for the safe, rapid, and persistent manufacture of recombinant T cells. Sci Adv. (2021) 7:eabf1333. doi: 10.1126/sciadv.abf1333 33853779 PMC8046366

[78] Athanasopoulos T Munye MM Yáñez-Muñoz RJ. Nonintegrating gene therapy vectors. Hematol Oncol Clin North Am. (2017) 31:753–70. doi: 10.1016/j.hoc.2017.06.007 28895845

[79] Yew CHT Gurumoorthy N Nordin F Tye GJ Wan Kamarul Zaman WS Tan JJ. Integrase deficient lentiviral vector: prospects for safe clinical applications. PeerJ. (2022) 10:e13704. doi: 10.7717/peerj.13704 35979475 PMC9377332

[80] Yoon SH Lee JM Cho HI Kim EK Kim HS Park MY. Adoptive immunotherapy using human peripheral blood lymphocytes transferred with RNA encoding Her-2/neu-specific chimeric immune receptor in ovarian cancer xenograft model. Cancer Gene Ther. (2009) 16:489–97. doi: 10.1038/cgt.2008.98 19096447

[81] Zhao Y Zheng Z Cohen CJ Gattinoni L Palmer DC Restifo NP. High-efficiency transfection of primary human and mouse T lymphocytes using RNA electroporation. Mol Ther. (2006) 13:151–9. doi: 10.1016/j.ymthe.2005.07.688 PMC1473967 16140584

[82] Beatty GL Haas AR Maus MV Torigian DA Soulen MC Plesa G. Mesothelin-specific chimeric antigen receptor mRNA-engineered T cells induce antitumor activity in solid Malignancies. Cancer Immunol Res. (2014) 2:112–20. doi: 10.1158/2326-6066.CIR-13-0170 PMC3932715 24579088

[83] Zhao Y Moon E Carpenito C Paulos CM Liu X Brennan AL. Multiple injections of electroporated autologous T cells expressing a chimeric antigen receptor mediate regression of human disseminated tumor. Cancer Res. (2010) 70:9053–61. doi: 10.1158/0008-5472.CAN-10-2880 PMC2982929 20926399

[84] Dunbar CE High KA Joung JK Kohn DB Ozawa K Sadelain M. Gene therapy comes of age. Science. (2018) 359:eaan4672. doi: 10.1126/science.aan4672 29326244

[85] Michels A Ho N Buchholz CJ. Precision medicine: *In vivo* CAR therapy as a showcase for receptor-targeted vector platforms. Mol Ther. (2022) 30:2401–15. doi: 10.1016/j.ymthe.2022.05.018 PMC9263322 35598048

[86] Anastasov N Höfig I Mall S Krackhardt AM Thirion C. Optimized Lentiviral Transduction Protocols by Use of a Poloxamer Enhancer, Spinoculation, and scFv-Antibody Fusions to VSV-G. In: Federico M, editor. Lentiviral Vectors and Exosomes as Gene and Protein Delivery Tools. Springer New York, New York, NY (2016). p. 49–61. doi: 10.1007/978-1-4939-3753-0_4 27317172

[87] Yu B Shi Q Belk JA Yost KE Parker KR Li R. Engineered cell entry links receptor biology with single-cell genomics. Cell. (2022) 185:4904–4920.e22. doi: 10.1016/j.cell.2022.11.016 36516854 PMC9789208

[88] Buchholz CJ Mühlebach MD Cichutek K. Lentiviral vectors with measles virus glycoproteins – dream team for gene transfer? Trends Biotechnol. (2009) 27:259–65. doi: 10.1016/j.tibtech.2009.02.002 19327858

[89] Nakamura T Peng KW Harvey M Greiner S Lorimer IAJ James CD. Rescue and propagation of fully retargeted oncolytic measles viruses. Nat Biotechnol. (2005) 23:209–14. doi: 10.1038/nbt1060 15685166

[90] Charitidis FT Adabi E Thalheimer FB Clarke C Buchholz CJ. Monitoring CAR T cell generation with a CD8-targeted lentiviral vector by single-cell transcriptomics. Mol Ther - Methods Clin Dev. (2021) 23:359–69. doi: 10.1016/j.omtm.2021.09.019 PMC8546366 34729382

[91] Samulski RJ Muzyczka N. AAV-mediated gene therapy for research and therapeutic purposes. Annu Rev Virol. (2014) 1:427–51. doi: 10.1146/annurev-virology-031413-085355 26958729

[92] Michels A Frank AM Günther DM Mataei M Börner K Grimm D. Lentiviral and adeno-associated vectors efficiently transduce mouse T lymphocytes when targeted to murine CD8. Mol Ther - Methods Clin Dev. (2021) 23:334–47. doi: 10.1016/j.omtm.2021.09.014 PMC8531454 34729380

[93] Nawaz W Huang B Xu S Li Y Zhu L Yiqiao H. AAV-mediated in *vivo* CAR gene therapy for targeting human T-cell leukemia. Blood Cancer J. (2021) 11:119. doi: 10.1038/s41408-021-00508-1 34162832 PMC8222347

[94] Kitte R Rabel M Geczy R Park S Fricke S Koehl U. Lipid nanoparticles outperform electroporation in mRNA-based CAR T cell engineering. Mol Ther - Methods Clin Dev. (2023) 31:101139. doi: 10.1016/j.omtm.2023.101139 38027056 PMC10663670

[95] Smith TT Stephan SB Moffett HF McKnight LE Ji W Reiman D. *In situ* programming of leukaemia-specific T cells using synthetic DNA nanocarriers. Nat Nanotechnol. (2017) 12:813–20. doi: 10.1038/nnano.2017.57 PMC5646367 28416815

[96] Rurik JG Tombácz I Yadegari A Méndez Fernández PO Shewale SV Li L. CAR T cells produced in *vivo* to treat cardiac injury. Science. (2022) 375:91–6. doi: 10.1126/science.abm0594 PMC9983611 34990237

[97] Parayath NN Stephan SB Koehne AL Nelson PS Stephan MT. *In vitro*-transcribed antigen receptor mRNA nanocarriers for transient expression in circulating T cells in *vivo* . Nat Commun. (2020) 11:6080. doi: 10.1038/s41467-020-19486-2 33247092 PMC7695830

[98] Parhiz H Shuvaev VV Pardi N Khoshnejad M Kiseleva RY Brenner JS. PECAM-1 directed re-targeting of exogenous mRNA providing two orders of magnitude enhancement of vascular delivery and expression in lungs independent of apolipoprotein E-mediated uptake. J Controlled Release. (2018) 291:106–15. doi: 10.1016/j.jconrel.2018.10.015 PMC6477695 30336167

[99] Tombácz I Laczkó D Shahnawaz H Muramatsu H Natesan A Yadegari A. Highly efficient CD4+ T cell targeting and genetic recombination using engineered CD4+ cell-homing mRNA-LNPs. Mol Ther. (2021) 29:3293–304. doi: 10.1016/j.ymthe.2021.06.004 PMC8571164 34091054

[100] Munis AM Mattiuzzo G Bentley EM Collins MK Eyles JE Takeuchi Y. Use of heterologous vesiculovirus G proteins circumvents the humoral anti-envelope immunity in lentivector-based *in vivo* gene delivery. Mol Ther - Nucleic Acids. (2019) 17:126–37. doi: 10.1016/j.omtn.2019.05.010 PMC6599914 31254925

[101] Weber T. Anti-AAV antibodies in AAV gene therapy: current challenges and possible solutions. Front Immunol. (2021) 12:658399/full. doi: 10.3389/fimmu.2021.658399/full 33815421 PMC8010240

[102] Maude SL Laetsch TW Buechner J Rives S Boyer M Bittencourt H. Tisagenlecleucel in children and young adults with B-cell lymphoblastic leukemia. N Engl J Med. (2018) 378:439–48. doi: 10.1056/NEJMoa1709866 PMC5996391 29385370

[103] Munshi NC Anderson LD Shah N Madduri D Berdeja J Lonial S. Idecabtagene vicleucel in relapsed and refractory multiple myeloma. N Engl J Med. (2021) 384:705–16. doi: 10.1056/NEJMoa2024850 33626253

[104] Park JH Rivière I Gonen M Wang X Sénéchal B Curran KJ. Long-term follow-up of CD19 CAR therapy in acute lymphoblastic leukemia. N Engl J Med. (2018) 378:449–59. doi: 10.1056/NEJMoa1709919 PMC6637939 29385376

[105] Raje N Berdeja J Lin Y Siegel D Jagannath S Madduri D. Anti-BCMA CAR T-cell therapy bb2121 in relapsed or refractory multiple myeloma. N Engl J Med. (2019) 380:1726–37. doi: 10.1056/NEJMoa1817226 PMC8202968 31042825

[106] Schuster SJ Bishop MR Tam CS Waller EK Borchmann P McGuirk JP. Tisagenlecleucel in adult relapsed or refractory diffuse large B-cell lymphoma. N Engl J Med. (2019) 380:45–56. doi: 10.1056/NEJMoa1804980 30501490

[107] Wang M Munoz J Goy A Locke FL Jacobson CA Hill BT. KTE-X19 CAR T-cell therapy in relapsed or refractory mantle-cell lymphoma. N Engl J Med. (2020) 382:1331–42. doi: 10.1056/NEJMoa1914347 PMC7731441 32242358

[108] Wagner J Wickman E DeRenzo C Gottschalk S. CAR T cell therapy for solid tumors: bright future or dark reality? Mol Ther. (2020) 28:2320–39. doi: 10.1016/j.ymthe.2020.09.015 PMC7647674 32979309

[109] Patel U Abernathy J Savani BN Oluwole O Sengsayadeth S Dholaria B. CAR T cell therapy in solid tumors: A review of current clinical trials. eJHaem. (2022) 3:24–31. doi: 10.1002/jha2.356 35844304 PMC9175685

[110] Rad SMAH Halpin JC Mollaei M Smith Bell SWJ Hirankarn N McLellan AD. Metabolic and mitochondrial functioning in chimeric antigen receptor (CAR)—T cells. Cancers. (2021) 13:1229. doi: 10.3390/cancers13061229 33799768 PMC8002030

[111] Hou AJ Chen LC Chen YY. Navigating CAR-T cells through the solid-tumour microenvironment. Nat Rev Drug Discovery. (2021) 20:531–50. doi: 10.1038/s41573-021-00189-2 33972771

[112] McLellan AD Ali Hosseini Rad SM. Chimeric antigen receptor T cell persistence and memory cell formation. Immunol Cell Biol. (2019) 97:664–74. doi: 10.1111/imcb.12254 31009109

[113] MacLeod DT Antony J Martin AJ Moser RJ Hekele A Wetzel KJ. Integration of a CD19 CAR into the TCR alpha chain locus streamlines production of allogeneic gene-edited CAR T cells. Mol Ther. (2017) 25:949–61. doi: 10.1016/j.ymthe.2017.02.005 PMC5383629 28237835

[114] Poirot L Philip B Schiffer-Mannioui C Le Clerre D Chion-Sotinel I Derniame S. Multiplex genome-edited T-cell manufacturing platform for “Off-the-shelf” Adoptive T-cell immunotherapies. Cancer Res. (2015) 75:3853–64. doi: 10.1158/0008-5472.CAN-14-3321 26183927

[115] Qasim W Zhan H Samarasinghe S Adams S Amrolia P Stafford S. Molecular remission of infant B-ALL after infusion of universal TALEN gene-edited CAR T cells. Sci Transl Med. (2017) 9:eaaj2013. doi: 10.1126/scitranslmed.aaj2013 28123068

[116] Osborn MJ Webber BR Knipping F Lonetree Cl Tennis N DeFeo AP. Evaluation of TCR gene editing achieved by TALENs, CRISPR/cas9, and megaTAL nucleases. Mol Ther. (2016) 24:570–81. doi: 10.1038/mt.2015.197 PMC4786913 26502778

[117] Tao R Han X Bai X Yu J Ma Y Chen W. Revolutionizing cancer treatment: enhancing CAR-T cell therapy with CRISPR/Cas9 gene editing technology. Front Immunol. (2024) 15:1354825/full. doi: 10.3389/fimmu.2024.1354825/full 38449862 PMC10914996

[118] Symington LS Gautier J. Double-strand break end resection and repair pathway choice. Annu Rev Genet. (2011) 45:247–71. doi: 10.1146/annurev-genet-110410-132435 21910633

[119] Anzalone AV Koblan LW Liu DR. Genome editing with CRISPR–Cas nucleases, base editors, transposases and prime editors. Nat Biotechnol. (2020) 38:824–44. doi: 10.1038/s41587-020-0561-9 32572269

[120] Biederstädt A Manzar GS Daher M. Multiplexed engineering and precision gene editing in cellular immunotherapy. Front Immunol. (2022) 13:1063303/full. doi: 10.3389/fimmu.2022.1063303/full 36483551 PMC9723254

[121] Dimitri A Herbst F Fraietta JA. Engineering the next-generation of CAR T-cells with CRISPR-Cas9 gene editing. Mol Cancer. (2022) 21:78. doi: 10.1186/s12943-022-01559-z 35303871 PMC8932053

[122] McCarty NS Graham AE Studená L Ledesma-Amaro R. Multiplexed CRISPR technologies for gene editing and transcriptional regulation. Nat Commun. (2020) 11:1281. doi: 10.1038/s41467-020-15053-x 32152313 PMC7062760

[123] Lonez C Breman E. Allogeneic CAR-T therapy technologies: has the promise been met? Cells. (2024) 13:146. doi: 10.3390/cells13020146 38247837 PMC10814647

[124] Tycko J Wainberg M Marinov GK Ursu O Hess GT Ego BK. Mitigation of off-target toxicity in CRISPR-Cas9 screens for essential non-coding elements. Nat Commun. (2019) 10:4063. doi: 10.1038/s41467-019-11955-7 31492858 PMC6731277

[125] Morgens DW Wainberg M Boyle EA Ursu O Araya CL Tsui CK. Genome-scale measurement of off-target activity using Cas9 toxicity in high-throughput screens. Nat Commun. (2017) 8:15178. doi: 10.1038/ncomms15178 28474669 PMC5424143

[126] Pattanayak V Lin S Guilinger JP Ma E Doudna JA Liu DR. High-throughput profiling of off-target DNA cleavage reveals RNA-programmed Cas9 nuclease specificity. Nat Biotechnol. (2013) 31:839–43. doi: 10.1038/nbt.2673 PMC3782611 23934178

[127] Fu Y Sander JD Reyon D Cascio VM Joung JK. Improving CRISPR-Cas nuclease specificity using truncated guide RNAs. Nat Biotechnol. (2014) 32:279–84. doi: 10.1038/nbt.2808 PMC3988262 24463574

[128] Ran FA Hsu PD Lin CY Gootenberg JS Konermann S Trevino AE. Double nicking by RNA-guided CRISPR cas9 for enhanced genome editing specificity. Cell. (2013) 154:1380–9. doi: 10.1016/j.cell.2013.08.021 PMC3856256 23992846

[129] Guilinger JP Thompson DB Liu DR. Fusion of catalytically inactive Cas9 to FokI nuclease improves the specificity of genome modification. Nat Biotechnol. (2014) 32:577–82. doi: 10.1038/nbt.2909 PMC4263420 24770324

[130] Tsai SQ Wyvekens N Khayter C Foden JA Thapar V Reyon D. Dimeric CRISPR RNA-guided FokI nucleases for highly specific genome editing. Nat Biotechnol. (2014) 32:569–76. doi: 10.1038/nbt.2908 PMC4090141 24770325

[131] Lin S Staahl BT Alla RK Doudna JA. Enhanced homology-directed human genome engineering by controlled timing of CRISPR/Cas9 delivery. eLife. (2014) 3:e04766. doi: 10.7554/eLife.04766 25497837 PMC4383097

[132] Kim S Kim D Cho SW Kim J Kim JS. Highly efficient RNA-guided genome editing in human cells *via* delivery of purified Cas9 ribonucleoproteins. Genome Res. (2014) 24:1012–9. doi: 10.1101/gr.171322.113 PMC4032847 24696461

[133] Ren J Zhang X Liu X Fang C Jiang S June CH. A versatile system for rapid multiplex genome-edited CAR T cell generation. Oncotarget. (2017) 8:17002–11. doi: 10.18632/oncotarget.15218 PMC5370017 28199983

[134] Ren J Liu X Fang C Jiang S June CH Zhao Y. Multiplex genome editing to generate universal CAR T cells resistant to PD1 inhibition. Clin Cancer Res. (2017) 23:2255–66. doi: 10.1158/1078-0432.CCR-16-1300 PMC5413401 27815355

[135] Zou F Lu L Liu J Xia B Zhang W Hu Q. Engineered triple inhibitory receptor resistance improves anti-tumor CAR-T cell performance *via* CD56. Nat Commun. (2019) 10:4109. doi: 10.1038/s41467-019-11893-4 31511513 PMC6739330

[136] Lee YH Lee HJ Kim HC Lee Y Nam SK Hupperetz C. PD-1 and TIGIT downregulation distinctly affect the effector and early memory phenotypes of CD19-targeting CAR T cells. Mol Ther. (2022) 30:579–92. doi: 10.1016/j.ymthe.2021.10.004 PMC8821960 34628052

[137] Ciraolo E Althoff S Ruß J Rosnev S Butze M Pühl M. Simultaneous genetic ablation of PD-1, LAG-3, and TIM-3 in CD8 T cells delays tumor growth and improves survival outcome. Int J Mol Sci. (2022) 23:1–14. doi: 10.3390/ijms23063207 PMC8955581 35328630

[138] Kurata M Wolf NK Lahr WS Weg MT Kluesner MG Lee S. Highly multiplexed genome engineering using CRISPR/Cas9 gRNA arrays. PloS One. (2018) 13:e0198714. doi: 10.1371/journal.pone.0198714 30222773 PMC6141065

[139] Zhang S Voigt CA. Engineered dCas9 with reduced toxicity in bacteria: implications for genetic circuit design. Nucleic Acids Res. (2018) 46:11115–25. doi: 10.1093/nar/gky884/5115820 PMC6237744 30289463

[140] Nowak CM Lawson S Zerez M Bleris L. Guide RNA engineering for versatile Cas9 functionality. Nucleic Acids Res. (2016) 44:9555–64. doi: 10.1093/nar/gkw908 PMC5175371 27733506

[141] Adikusuma F Piltz S Corbett MA Turvey M McColl SR Helbig KJ. Large deletions induced by Cas9 cleavage. Nature. (2018) 560:E8–9. doi: 10.1038/s41586-018-0380-z 30089922

[142] Papathanasiou S Markoulaki S Blaine LJ Leibowitz ML Zhang CZ Jaenisch R. Whole chromosome loss and genomic instability in mouse embryos after CRISPR-Cas9 genome editing. Nat Commun. (2021) 12:5855. doi: 10.1038/s41467-021-26097-y 34615869 PMC8494802

[143] Alanis-Lobato G Zohren J McCarthy A Fogarty NME Kubikova N Hardman E. Frequent loss of heterozygosity in CRISPR-Cas9–edited early human embryos. Proc Natl Acad Sci. (2021) 118:e2004832117. doi: 10.1073/pnas.2004832117 34050011 PMC8179174

[144] Weisheit I Kroeger JA Malik R Klimmt J Crusius D Dannert A. Detection of deleterious on-target effects after HDR-mediated CRISPR editing. Cell Rep. (2020) 31:107689. doi: 10.1016/j.celrep.2020.107689 32460021

[145] Boutin J Rosier J Cappellen D Prat F Toutain J Pennamen P. CRISPR-Cas9 globin editing can induce megabase-scale copy-neutral losses of heterozygosity in hematopoietic cells. Nat Commun. (2021) 12:4922. doi: 10.1038/s41467-021-25190-6 34389729 PMC8363739

[146] Nahmad AD Reuveni E Goldschmidt E Tenne T Liberman M Horovitz-Fried M. Frequent aneuploidy in primary human T cells after CRISPR–Cas9 cleavage. Nat Biotechnol. (2022) 40:1807–13. doi: 10.1038/s41587-022-01377-0 PMC7613940 35773341

[147] Yen ST Zhang M Deng JM Usman SJ Smith CN Parker-Thornburg J. Somatic mosaicism and allele complexity induced by CRISPR/Cas9 RNA injections in mouse zygotes. Dev Biol. (2014) 393:3–9. doi: 10.1016/j.ydbio.2014.06.017 24984260 PMC4166609

[148] Mehravar M Shirazi A Nazari M Banan M. Mosaicism in CRISPR/Cas9-mediated genome editing. Dev Biol. (2019) 445:156–62. doi: 10.1016/j.ydbio.2018.10.008 30359560

[149] Gaudelli NM Komor AC Rees HA Packer MS Badran AH Bryson DI. Programmable base editing of A•T to G•C in genomic DNA without DNA cleavage. Nature. (2017) 551:464–71. doi: 10.1038/nature24644 PMC5726555 29160308

[150] Komor AC Kim YB Packer MS Zuris JA Liu DR. Programmable editing of a target base in genomic DNA without double-stranded DNA cleavage. Nature. (2016) 533:420–4. doi: 10.1038/nature17946 PMC4873371 27096365

[151] Mok BY Kotrys AV Raguram A Huang TP Mootha VK Liu DR. CRISPR-free base editors with enhanced activity and expanded targeting scope in mitochondrial and nuclear DNA. Nat Biotechnol. (2022) 40:1378–87. doi: 10.1038/s41587-022-01256-8 PMC9463067 35379961

[152] Newby GA Yen JS Woodard KJ Mayuranathan T Lazzarotto CR Li Y. Base editing of haematopoietic stem cells rescues sickle cell disease in mice. Nature. (2021) 595:295–302. doi: 10.1038/s41586-021-03609-w 34079130 PMC8266759

[153] Han W Qiu HY Sun S Fu ZC Wang GQ Qian X. Base editing of the HBG promoter induces potent fetal hemoglobin expression with no detectable off-target mutations in human HSCs. Cell Stem Cell. (2023) 30:1624–39. doi: 10.1016/j.stem.2023.10.007 37989316

[154] Badat M Ejaz A Hua P Rice S Zhang W Hentges LD. Direct correction of haemoglobin E β-thalassaemia using base editors. Nat Commun. (2023) 14:2238. doi: 10.1038/s41467-023-37604-8 37076455 PMC10115876

[155] Musunuru K Chadwick AC Mizoguchi T Garcia SP DeNizio JE Reiss CW. *In vivo* CRISPR base editing of PCSK9 durably lowers cholesterol in primates. Nature. (2021) 593:429–34. doi: 10.1038/s41586-021-03534-y 34012082

[156] Georgiadis C Rasaiyaah J Gkazi SA Preece R Etuk A Christi A. Base-edited CAR T cells for combinational therapy against T cell Malignancies. Leukemia. (2021) 35:3466–81. doi: 10.1038/s41375-021-01282-6 PMC8632682 34035409

[157] Diorio C Murray R Naniong M Barrera L Camblin A Chukinas J. Cytosine base editing enables quadruple-edited allogeneic CART cells for T-ALL. Blood. (2022) 140:619–29. doi: 10.1182/blood.2022015825 PMC9373016 35560156

[158] Webber BR Lonetree Cl Kluesner MG Johnson MJ Pomeroy EJ Diers MD. Highly efficient multiplex human T cell engineering without double-strand breaks using Cas9 base editors. Nat Commun. (2019) 10:5222. doi: 10.1038/s41467-019-13007-6 31745080 PMC6864045

[159] Anzalone AV Randolph PB Davis JR Sousa AA Koblan LW Levy JM. Search-and-replace genome editing without double-strand breaks or donor DNA. Nature. (2019) 576:149–57. doi: 10.1038/s41586-019-1711-4 PMC6907074 31634902

[160] Nelson JW Randolph PB Shen SP Everette KA Chen PJ Anzalone AV. Engineered pegRNAs improve prime editing efficiency. Nat Biotechnol. (2022) 40:402–10. doi: 10.1038/s41587-021-01039-7 PMC8930418 34608327

[161] Rees HA Komor AC Yeh WH Caetano-Lopes J Warman M Edge ASB. Improving the DNA specificity and applicability of base editing through protein engineering and protein delivery. Nat Commun. (2017) 8:15790. doi: 10.1038/ncomms15790 28585549 PMC5467206

[162] Yeh WH Chiang H Rees HA Edge ASB Liu DR. *In vivo* base editing of post-mitotic sensory cells. Nat Commun. (2018) 9:2184. doi: 10.1038/s41467-018-04580-3 29872041 PMC5988727

[163] Kim K Ryu SM Kim ST Baek G Kim D Lim K. Highly efficient RNA-guided base editing in mouse embryos. Nat Biotechnol. (2017) 35:435–7. doi: 10.1038/nbt.3816 28244995

[164] Gaidukov L Wroblewska L Teague B Nelson T Zhang X Liu Y. A multi-landing pad DNA integration platform for mammalian cell engineering. Nucleic Acids Res. (2018) 46:4072–86. doi: 10.1093/nar/gky216 PMC5934685 29617873

[165] Yarnall MTN Ioannidi EI Schmitt-Ulms C Krajeski RN Lim J Villiger L. Drag-and-drop genome insertion of large sequences without double-strand DNA cleavage using CRISPR-directed integrases. Nat Biotechnol. (2023) 41:500–12. doi: 10.1038/s41587-022-01527-4 PMC10257351 36424489

[166] Peters JE Makarova KS Shmakov S Koonin EV. Recruitment of CRISPR-Cas systems by Tn7-like transposons. Proc Natl Acad Sci. (2017) 114:E7358–66. doi: 10.1073/pnas.1709035114 PMC5584455 28811374

[167] Vo PLH Ronda C Klompe SE Chen EE Acree C Wang HH. CRISPR RNA-guided integrases for high-efficiency, multiplexed bacterial genome engineering. Nat Biotechnol. (2021) 39:480–9. doi: 10.1038/s41587-020-00745-y PMC10583764 33230293

[168] Hoffmann FT Kim M Beh LY Wang J Vo PLH Gelsinger DR. Selective TnsC recruitment enhances the fidelity of RNA-guided transposition. Nature. (2022) 609:384–93. doi: 10.1038/s41586-022-05059-4 PMC10583602 36002573

[169] Cai R Lv R Shi X Yang G Jin J. CRISPR/dCas9 tools: epigenetic mechanism and application in gene transcriptional regulation. Int J Mol Sci. (2023) 24:14865. doi: 10.3390/ijms241914865 37834313 PMC10573330

[170] Han JL Entcheva E. Gene modulation with CRISPR-based tools in human iPSC-cardiomyocytes. Stem Cell Rev Rep. (2023) 19:886–905. doi: 10.1007/s12015-023-10506-4 36656467 PMC9851124

[171] Nuñez JK Chen J Pommier GC Cogan JZ Replogle JM Adriaens C. Genome-wide programmable transcriptional memory by CRISPR-based epigenome editing. Cell. (2021) 184:2503–19. doi: 10.1016/j.cell.2021.03.025 PMC8376083 33838111

[172] Schmidt R Steinhart Z Layeghi M Freimer JW Bueno R Nguyen VQ. CRISPR activation and interference screens decode stimulation responses in primary human T cells. Science. (2022) 375:eabj4008. doi: 10.1126/science.abj4008 35113687 PMC9307090

[173] Ye L Park JJ Peng L Yang Q Chow RD Dong MB. A genome-scale gain-of-function CRISPR screen in CD8 T cells identifies proline metabolism as a means to enhance CAR-T therapy. Cell Metab. (2022) 34:595–614.e14. doi: 10.1016/j.cmet.2022.02.009 35276062 PMC8986623

[174] Jensen TI Mikkelsen NS Gao Z Foßelteder J Pabst G Axelgaard E. Targeted regulation of transcription in primary cells using CRISPRa and CRISPRi. Genome Res. (2021) 31:2120–30. doi: 10.1101/gr.275607.121 PMC8559706 34407984

[175] Chavez A Scheiman J Vora S Pruitt BW Tuttle M Iyer E PR. Highly efficient Cas9-mediated transcriptional programming. Nat Methods. (2015) 12:326–8. doi: 10.1038/nmeth.3312 PMC4393883 25730490

[176] Matharu N Rattanasopha S Tamura S Maliskova L Wang Y Bernard A. CRISPR-mediated activation of a promoter or enhancer rescues obesity caused by haploinsufficiency. Science. (2019) 363:eaau0629. doi: 10.1126/science.aau0629 30545847 PMC6570489

[177] Savell KE Bach SV Zipperly ME Revanna JS Goska NA Tuscher JJ. A neuron-optimized CRISPR/dCas9 activation system for robust and specific gene regulation. eNeuro. (2019) 6:ENEURO.0495–18.2019. doi: 10.1523/ENEURO.0495-18.2019 PMC6412672 30863790

[178] Abudayyeh OO Gootenberg JS Konermann S Joung J Slaymaker IM Cox DBT. C2c2 is a single-component programmable RNA-guided RNA-targeting CRISPR effector. Science. (2016) 353:aaf5573. doi: 10.1126/science.aaf5573 27256883 PMC5127784

[179] Abudayyeh OO Gootenberg JS Essletzbichler P Han S Joung J Belanto JJ. RNA targeting with CRISPR–cas13. Nature. (2017) 550:280–4. doi: 10.1038/nature24049 PMC5706658 28976959

[180] Cox DBT Gootenberg JS Abudayyeh OO Franklin B Kellner MJ Joung J. RNA editing with CRISPR-cas13. Science. (2017) 358:1019–27. doi: 10.1126/science.aaq0180 PMC5793859 29070703

[181] Smargon AA Cox DBT Pyzocha NK Zheng K Slaymaker IM Gootenberg JS. Cas13b is a type VI-B CRISPR-associated RNA-guided RNase differentially regulated by accessory proteins csx27 and csx28. Mol Cell. (2017) 65:618–630.e7. doi: 10.1016/j.molcel.2016.12.023 28065598 PMC5432119

[182] Shmakov S Abudayyeh OO Makarova KS Wolf YI Gootenberg JS Semenova E. Discovery and functional characterization of diverse class 2 CRISPR-cas systems. Mol Cell. (2015) 60:385–97. doi: 10.1016/j.molcel.2015.10.008 PMC4660269 26593719

[183] Konermann S Lotfy P Brideau NJ Oki J Shokhirev MN Hsu PD. Transcriptome engineering with RNA-targeting type VI-D CRISPR effectors. Cell. (2018) 173:665–76. doi: 10.1016/j.cell.2018.02.033 PMC5910255 29551272

[184] Jing X Xie B Chen L Zhang N Jiang Y Qin H. Implementation of the CRISPR-Cas13a system in fission yeast and its repurposing for precise RNA editing. Nucleic Acids Res. (2018) 46:e90–0. doi: 10.1093/nar/gky433 PMC6125684 29860393

[185] Huynh N Depner N Larson R King-Jones K. A versatile toolkit for CRISPR-Cas13-based RNA manipulation in Drosophila. Genome Biol. (2020) 21:279. doi: 10.1186/s13059-020-02193-y 33203452 PMC7670108

[186] He B Peng W Huang J Zhang H Zhou Y Yang X. Modulation of metabolic functions through Cas13d-mediated gene knockdown in liver. Protein Cell. (2020) 11:518–24. doi: 10.1007/s13238-020-00700-2 PMC7095259 32185621

[187] Kushawah G Hernandez-Huertas L Abugattas-Nuñez del Prado J Martinez-Morales JR DeVore ML Hassan H. CRISPR-cas13d induces efficient mRNA knockdown in animal embryos. Dev Cell. (2020) 54:805–817.e7. doi: 10.1016/j.devcel.2020.07.013 32768421

[188] Zhou H Su J Hu X Zhou C Li H Chen Z. Glia-to-neuron conversion by CRISPR-casRx alleviates symptoms of neurological disease in mice. Cell. (2020) 181:590–603.e16. doi: 10.1016/j.cell.2020.03.024 32272060

[189] Powell JE Lim CKW Krishnan R McCallister TX Saporito-Magriña C Zeballos MA. Targeted gene silencing in the nervous system with CRISPR-Cas13. Sci Adv. (2022) 8:eabk2485. doi: 10.1126/sciadv.abk2485 35044815 PMC8769545

[190] Freije CA Myhrvold C Boehm CK Lin AE Welch NL Carter A. Programmable inhibition and detection of RNA viruses using cas13. Mol Cell. (2019) 76:826–837.e11. doi: 10.1016/j.molcel.2019.09.013 31607545 PMC7422627

[191] Blanchard EL Vanover D Bawage SS Tiwari PM Rotolo L Beyersdorf J. Treatment of influenza and SARS-CoV-2 infections *via* mRNA-encoded Cas13a in rodents. Nat Biotechnol. (2021) 39:717–26. doi: 10.1038/s41587-021-00822-w 33536629

[192] Bot JF van der Oost J Geijsen N. The double life of CRISPR–Cas13. Curr Opin Biotechnol. (2022) 78:102789. doi: 10.1016/j.copbio.2022.102789 36115160

[193] Schwarz DS Hutvágner G Du T Xu Z Aronin N Zamore PD. Asymmetry in the assembly of the RNAi enzyme complex. Cell. (2003) 115:199–208. doi: 10.1016/S0092-8674(03)00759-1 14567917

[194] Wang Y Luo J Zhang H Lu J. microRNAs in the same clusters evolve to coordinately regulate functionally related genes. Mol Biol Evol. (2016) 33:2232–47. doi: 10.1093/molbev/msw089 PMC4989102 27189568

[195] Winter J Jung S Keller S Gregory RI Diederichs S. Many roads to maturity: microRNA biogenesis pathways and their regulation. Nat Cell Biol. (2009) 11:228–34. doi: 10.1038/ncb0309-228 19255566

[196] Rossi M Steklov M Huberty F Nguyen T Marijsse J Jacques-Hespel C. Efficient shRNA-based knockdown of multiple target genes for cell therapy using a chimeric miRNA cluster platform. Mol Ther - Nucleic Acids. (2023) 34:102038. doi: 10.1016/j.omtn.2023.102038 37799328 PMC10548280

[197] Boudreau RL Martins I Davidson BL. Artificial MicroRNAs as siRNA Shuttles: Improved Safety as Compared to shRNAs In vitro and *In vivo* . Mol Ther. (2009) 17:169–75. doi: 10.1038/mt.2008.231 PMC2834985 19002161

[198] Grimm D. The dose can make the poison: lessons learned from adverse in *vivo* toxicities caused by RNAi overexpression. Silence. (2011) 2:8. doi: 10.1186/1758-907X-2-8 22029761 PMC3234190

[199] Maczuga P Lubelski J van Logtenstein R Borel F Blits B Fakkert E. Embedding siRNA sequences targeting Apolipoprotein B100 in shRNA and miRNA scaffolds results in differential processing and in *vivo* efficacy. Mol Ther. (2013) 21:217–27. doi: 10.1038/mt.2012.160 PMC3538299 23089734

[200] Zhang P Ying P Li H Zhao N Liu R Li S. A novel safer CD19CAR with shRNA interference of IFN-γ can reduce multiple cytokine levels without significantly compromising its killing efficacy. Apoptosis. (2024) 29:556–67. doi: 10.1007/s10495-023-01925-2 38114800

[201] Shang S Chen Y Yang X Yang Y Wang W Wang Y. RNA silencing of GM-CSF in CAR-T cells reduces the secretion of multiple inflammatory cytokines. Invest New Drugs. (2023) 41:220–5. doi: 10.1007/s10637-023-01344-9 PMC10050814 36988829

[202] Jafarzadeh L Masoumi E Mirzaei HR Alishah K Fallah-Mehrjardi K Khakpoor-Koosheh M. Targeted knockdown of Tim3 by short hairpin RNAs improves the function of anti-mesothelin CAR T cells. Mol Immunol. (2021) 139:1–9. doi: 10.1016/j.molimm.2021.06.007 34450537

[203] Liu G Zhang Q Liu G Li D Zhang L Gu Z. Disruption of adenosine 2A receptor improves the anti-tumor function of anti-mesothelin CAR T cells both in vitro and in *vivo* . Exp Cell Res. (2021) 409:112886. doi: 10.1016/j.yexcr.2021.112886 34673000

[204] Altuvia Y. Clustering and conservation patterns of human microRNAs. Nucleic Acids Res. (2005) 33:2697–706. doi: 10.1093/nar/gki567 PMC1110742 15891114

[205] Bourhill T Arbuthnot P Ely A. Successful disabling of the 5′ UTR of HCV using adeno-associated viral vectors to deliver modular multimeric primary microRNA mimics. J Virol Methods. (2016) 235:26–33. doi: 10.1016/j.jviromet.2016.05.008 27181212

[206] Liu YP Haasnoot J ter Brake O Berkhout B Konstantinova P. Inhibition of HIV-1 by multiple siRNAs expressed from a single microRNA polycistron. Nucleic Acids Res. (2008) 36:2811–24. doi: 10.1093/nar/gkn109 PMC2396423 18346971

[207] Choi JG Bharaj P Abraham S Ma H Yi G Ye C. Multiplexing Seven miRNA-Based shRNAs to Suppress HIV Replication. Mol Ther. (2015) 23:310–20. doi: 10.1038/mt.2014.205 PMC4445613 25358251

[208] Boudreau RL Monteys AM Davidson BL. Minimizing variables among hairpin-based RNAi vectors reveals the potency of shRNAs. RNA. (2008) 14:1834–44. doi: 10.1261/rna.1062908 PMC2525944 18697922

[209] Rousset F Salmon P Bredl S Cherpin O Coelho M Myburgh R. Optimizing Synthetic miRNA Minigene Architecture for Efficient miRNA Hairpin Concatenation and Multi-target Gene Knockdown. Mol Ther - Nucleic Acids. (2019) 14:351–63. doi: 10.1016/j.omtn.2018.12.004 PMC6350225 30665184

[210] Yang S Im SH Chung JY Lee J Lee K Kang YK. An antibody-CRISPR/cas conjugate platform for target-specific delivery and gene editing in cancer. Adv Sci. (2024) e2308763. doi: 10.1002/advs.202308763 PMC11151032 38552157

[211] Leclerc D Siroky MD Miller SM. Next-generation biological vector platforms for in *vivo* delivery of genome editing agents. Curr Opin Biotechnol. (2024) 85:103040. doi: 10.1016/j.copbio.2023.103040 38103518

[212] Lee JH Han JP. *In vivo* LNP-CRISPR approaches for the treatment of hemophilia. Mol Diagn Ther. (2024) 28:239–48. doi: 10.1007/s40291-024-00705-1 PMC11068834 38538969

[213] Kerzel T Giacca G Beretta S Bresesti C Notaro M Scotti GM. *In vivo* macrophage engineering reshapes the tumor microenvironment leading to eradication of liver metastases. Cancer Cell. (2023) 41:1892–1910.e10. doi: 10.1016/j.ccell.2023.09.014 37863068

[214] Fu W Lei C Liu S Cui Y Wang C Qian K. CAR exosomes derived from effector CAR-T cells have potent antitumour effects and low toxicity. Nat Commun. (2019) 10:4355. doi: 10.1038/s41467-019-12321-3 31554797 PMC6761190

[215] Brocco D Lanuti P Simeone P Bologna G Pieragostino D Cufaro MC. Circulating cancer stem cell-derived extracellular vesicles as a novel biomarker for clinical outcome evaluation. J Oncol. (2019) 2019:1–13. doi: 10.1155/2019/5879616 PMC6885781 31827511

[216] Tang XJ Sun XY Huang KM Zhang L Yang ZS Zou DD. Therapeutic potential of CAR-T cell-derived exosomes: a cell-free modality for targeted cancer therapy. Oncotarget. (2015) 6:44179–90. doi: 10.18632/oncotarget.6175 PMC4792550 26496034

[217] Brocco D De Bellis D Di Marino P Simeone P Grassadonia A De Tursi M. High blood concentration of leukocyte-derived extracellular vesicles is predictive of favorable clinical outcomes in patients with pancreatic cancer: results from a multicenter prospective study. Cancers. (2022) 14:4748. doi: 10.3390/cancers14194748 36230671 PMC9562679

[218] Yang P Cao X Cai H Feng P Chen X Zhu Y. The exosomes derived from CAR-T cell efficiently target mesothelin and reduce triple-negative breast cancer growth. Cell Immunol. (2021) 360:104262. doi: 10.1016/j.cellimm.2020.104262 33373818

[219] Dingfelder J Aigner M Taubmann J Minopoulou I Park S Kaplan CD. Fully human anti-CD19 CAR T cells derived from systemic lupus erythematosus patients exhibit cytotoxicity with reduced inflammatory cytokine production. Transplant Cell Ther. (2024) 30:582.e1–582.e10. doi: 10.1016/j.jtct.2024.03.023 38548226

